# Supplementary material for: Linear Relationships between Partition Coefficients of Different Organic Compounds and Proteins in Aqueous Two-Phase Systems of Various Polymer and Ionic Compositions
Source: Polymers (Basel). 2020 Jun 29;12(7):1452. doi: 10.3390/polym12071452 (PMC7408505; doi:10.3390/polym12071452)
Supplement: Supplementary file 1 [file polymers-12-01452-s001.pdf]

Supplementary Material

Linear relationships between partition coefficients of different organic compounds and proteins in aqueous two-phase systems of various polymer and ionic compositions

Nuno R. da Silva[a], Luisa A. Ferreira[b], Pedro P. Madeira[c], José A. Teixeira[a], Vladimir N. Uversky[d,*], Boris Y. Zaslavsky[b,*]

[a]IBB—Institute for Biotechnology and Bioengineering, Centre of Biological Engineering, Universidade do Minho, Campus de Gualtar,4710-057 Braga, Portugal;

[b]Cleveland Daignostics, 3615 Superior Ave., Cleveland, OH 44114, USA;

[c]Centro de Investigacao em Materiais Ceramicos e Compositos, Department of Chemistry, Aveiro, Portugal;

[d]Department of Molecular Medicine, Morsani College of Medicine, University of South Florida, Tampa, Florida 33612, USA

This PDF file includes:

Materials and Methods

ESI References

Tables S1 to S4

Table S1. Partition coefficients and logarithms of partition coefficients for small organic compounds in aqueous two-phase systems of various compositions (NaPB – sodium phosphate buffer, K/NaPB – sodium/potassium phosphate buffer)

Table S2. Partition coefficients and logarithms of partition coefficients for various proteins in aqueous two-phase systems of various compositions (NaPB – sodium phosphate buffer, K/NaPB – sodium/potassium phosphate buffer)

Table S3. Logarithms of distribution coefficients for various drugs in octanol-buffer systems of various ionic compositions (NaPB – sodium phosphate buffer, UB - Universal buffer composed of 0.01M or 0.10M each of phosphoric, boric, and acetic acids adjusted to pH 7.4 with NaOH)

Table S4. Logarithms of partition coefficients of drugs between blood and various tissues in rats *in vivo* (data from [S16])

References

S1. N. da Silva, L.A. Ferreira, P.P. Madeira, J.A. Teixeira, V.N. Uversky, B.Y. Zaslavsky, Effect of sodium chloride on solute-solvent interactions in aqueous polyethylene glycol-sodium sulfate two-phase systems, J. Chromatogr. A, 1425 (2015) 51-61.

S2. N. R. da Silva, L. A. Ferreira, J. A. Teixeira, V. N. Uversky, B. Y. ZaslavskyL. A. Effects of sodium chloride and sodium perchlorate on properties and partition behavior of solutes in aqueous dextran-polyethylene glycol and polyethylene glycol-sodium sulfate two-phase systems, J. Chromatogr. A,, 1583 (2019) 28-38.

S3. Madeira, P.P., Bessa, A., Loureiro, J.A., Alvares-Ribeiro, L., Rodrigues, A.E., Zaslavsky, B.Y., Cooperativity between various types of polar solute-solvent interactions in aqueous media., J. Chromatogr. A, 1408 (2015), 108-117.

S4. N. da Silva, L.A. Ferreira, P.P. Madeira, J.A. Teixeira, V.N. Uversky, B.Y. Zaslavsky, Analysis of partitioning of organic compounds and proteins in aqueous polyethylene glycol-sodium sulfate aqueous two-phase systems in terms of solute-solvent interactions. J. Chromatogr. A 1415 (2015) 1-10.

S5. Ferreira, L.A., Teixeira, J. A., Mikheeva, L.M., Chait, A., Zaslavsky B. Y. Effect of salt additives on partition of nonionic solutes in aqueous PEG- sodium sulfate two-phase system, J. Chromatogr. A 1218, 5031-5039 (2011).

S6. da Silva, N., Ferreira, L.A., Mikheeva, L.M., Teixeira, J., Zaslavsky, B.Y. Origin of salt additive effect on solute partitioning in aqueous PEG- sodium sulfate two-phase system. J. Chromatogr. A, 1337, 3-8 (2014).

S7. L. A. Ferreira, Madeira, P.P., Uversky, V.N., Zaslavsky, B.Y., Analyzing the effects of protecting osmolytes on solute-water interactions by solvatochromic comparison method: I. Small organic compounds RSC Advances, 5, 59812-59822 2015.

S8. Madeira, P.P., Bessa, A., Teixeira, M. A., Ribeiro, L.A., Aires-Barros, M.R., Rodrigues, A.E., Chait. A., Zaslavsky, B.Y., Solvatochromic Relationship: Prediction of Distribution of Ionic Solutes in Aqueous Two-Phase Systems. J. Chromatogr. A, 1271 (1) 10-16 (2013).

S9. Madeira, P., Reis, C.A., Rodrigues, A.E., Mikheeva, L.M., Zaslavsky, B.Y., Solvent properties governing solute partitioning in polymer/polymer aqueous two-phase systems: Nonionic compounds, J. Phys. Chem. B, 114 (1), 457-462 (2010).

S10. Madeira, P., Teixeira J.A., Macedo, E.A., Mikheeva, L.M., Zaslavsky, B.Y., Correlations between distribution coefficients of various biomolecules in different polymer/polymer aqueous two-phase systems, Fluid Phase Equil., 267, 150-157 (2008).

S11. Ferreira, L.A.,Madeira, P., Mikheeva, L.M., Uversky, V., Zaslavsky, B., Effect of salt additives on protein partition in polyethylene glycol-sodium sulfate aqueous two-phase systems. Biochim. Biophys. Acta, Proteins Proteomics,1834, 2859-2866 (2013).

S12. L.A. Ferreira, Zhonghua Wu, Lukasz Kurgan, V.N. Uversky, B.Y. Zaslavsky, How to manipulate partition behavior of proteins in aqueous two-phase system: Effect of trimethylamine N-oxide, Fluid Phase Equilibria, 2017, 449, 217-224.

S13. Madeira, P., Teixeira J.A., Macedo, E.A., Mikheeva, L.M., Zaslavsky, B.Y., "On the Collander equation": Protein partitioning in polymer/polymer aqueous two-phase systems, J.Chromatogr. A, 1190, 39-43 (2008).

S14. Ferreira L., Fan, X., Mikheeva, L.M., Madeira, P.P., Kurgan, L., Uversky, V.N., Zaslavsky, B.Y., Structural features important for differences in protein partitioning in aqueous dextran-polyethylene glycol two-phase systems of different ionic composition. Biochim. Biophys. Acta, Proteins Proteomics, 1844 (3) 694-704 (2014)

S15. L. A. Ferreira, X. Fan, Madeira, P.P., L. Kurgan, V. N. Uversky, B.Y. Zaslavsky, Analyzing the effects of protecting osmolytes on solute-water interactions by solvatochromic comparison method: II. Globular proteins. RSC Advances, 5, 59780-59791, 2015.

S16. P. Paixao, N. Aniceto, L.F. Gouveia, J.A. Morais, Tissue-to-blood distribution coefficients in the rat: utility for estimation of the volume of distribution in man, Eur J Pharm Sci, 50 (2013) 526-543.

Table S1. Partition coefficients and logarithms of partition coefficients for small organic compounds in aqueous two-phase systems of various compositions (NaPB – sodium phosphate buffer, K/NaPB – sodium/potassium phosphate buffer)

Ref	# ATPS	Polymer-1	Polymer-2	Salt	salt additive	Buffer	pH	Osmolyte	DNP-Ala	DNP-n-val	DNP-n-leu	DNP-AO	4-Aminophenol	Benzyl alcohol	Caffeine	Coumarin	Glucoside	Phenol	Methyl anthranilate	2-Phenylethanol	Vanillin	Adenine	AMP	ADP	ATP	Adenosine
S1	1	11.10% PEG-8000		6.33% Na2SO4	0.215M NaCl	0.01 M NaPB	6.8		4.43	5.43	7.15	13.93		4.067	2.11	4.86	2.47	6.5	9.59	4.98	8.28					
S1	2	11.10% PEG-10000		6.33% Na2SO4	0.215M NaCl	0.01 M NaPB	6.8		3.99	5.36	6.88	14.01		4.146	2.356	4.55	2.916	6.54	9.58	5.05	8.31					
S1	3	11.10% PEG-8000		6.33% Na2SO4	0.215M NaCl	0.01 M NaPB	6.8	0.5 M sorbitol	5.12	7.68	10.23	23.6		6.08	2.72	8.23	3.957	10.6	14.8	7.39	13.03					
S1	4	11.10% PEG-8000		6.33% Na2SO4	0.215M NaCl	0.01 M NaPB	6.8	0.5 M sucrose	5.09	7.87	10.81	25.04		6.61	2.55	8.46	4.05	11.97	16.42	8.52	13.22					
S1	5	11.10% PEG-8000		6.33% Na2SO4	0.215M NaCl	0.01 M NaPB	6.8	0.5 M TMAO	5.4	7.6	10.49	20.88		4.71	2.819	6.27	3.41	7.38	11.77	5.6	7.58					
S2	6	6.0% PEG-8000	12.0 Dex-75		0.215M NaCl	0.01 M K/NaPB	7.4		0.986	1.077	1.131	1.308		1.547	1.154	1.611	1.222	1.932	1.929	1.592	1.702					
S2	7	6.0% PEG-8000	12.0 Dex-75		0.215M NaCl	0.01 M K/NaPB	7.4	0.5 M sorbitol	1.1	1.227	1.3	1.557		1.63	1.176	1.742	1.31	2.116	2.135	1.779	1.939					
S2	8	6.0% PEG-8000	12.0 Dex-75		0.215M NaCl	0.01 M K/NaPB	7.4	0.5 M sucrose	1.144	1.251	1.346	1.624		1.722	1.184	1.777	1.35	2.234	2.218	1.846	1.987					
S2	9	6.0% PEG-8000	12.0 Dex-75		0.215M NaCl	0.01 M K/NaPB	7.4	0.5 M TMAO	1	1.107	1.17	1.372		1.563	1.208	1.66	1.26	1.906	1.932	1.667	1.641					
S2	10	6.0% PEG-8000	12.0 Dex-75		0.215M NaCl	0.01 M K/NaPB	7.4	1.5 M TMAO	1.11	1.271	1.406	1.722		1.554	1.311	1.923	1.36	1.998	2.467	1.701	1.818					
S2	11	6.0% PEG-8000	12.0 Dex-75		0.215M NaClO4	0.01 M K/NaPB	7.4		0.959	1.029	1.092	1.249		1.62	1.197	1.669	1.249	1.909	1.985	1.663	1.653					
S2	12	6.0% PEG-8000	12.0 Dex-75		0.215M NaClO4	0.01 M K/NaPB	7.4	0.5 M sorbitol	0.966	1.064	1.154	1.393		1.767	1.266	1.974	1.396	1.233	2.453	1.895	1.973					
S2	13	6.0% PEG-8000	12.0 Dex-75		0.215M NaClO4	0.01 M K/NaPB	7.4	0.5 M sucrose	0.971	1.074	1.17	1.419		1.857	1.246	1.977	1.409	2.396	2.581	2.035	2.047					
S2	14	6.0% PEG-8000	12.0 Dex-75		0.215M NaClO4	0.01 M K/NaPB	7.4	0.5 M TMAO	0.965	1.059	1.142	1.361		1.586	1.252	1.781	1.303	1.939	2.13	1.726	1.591					
S2	15	6.0% PEG-8000	12.0 Dex-75		0.215M NaClO4	0.01 M K/NaPB	7.4	1.5 M TMAO	0.994	1.175	1.346	1.854		1.855	1.432	2.445	1.515	2.265	3.099	2.034	1.778					
S2	16	11.10% PEG-8000		6.33% Na2SO4	0.215M NaClO4	0.01 M NaPB	6.8		3.511	5.19	7.15	13.9		6.08	2.582	10.74	2.897	9.92	18.6	8.19	12.47					
S2	17	11.10% PEG-10000		6.33% Na2SO4	0.215M NaClO4	0.01 M NaPB	6.8		3.67	5.36	7.24	15		5.8	2.99	10.3	3.3	8.63	15.9	7.33	12.4					
S2	18	11.10% PEG-8000		6.33% Na2SO4	0.215M NaClO4	0.01 M NaPB	6.8	0.5 M sorbitol	4.02	6.1	8.61	19.1		8	3.5	15.2	4.15	11.8	29.5	9.45	19.1					
S2	19	11.10% PEG-8000		6.33% Na2SO4	0.215M NaClO4	0.01 M NaPB	6.8	0.5 M sucrose	3.485	5.4	7.73	17.73		6.4	2.938	12.08	3.86	12.43	21.6	9.67	14.9					
S2	20	11.10% PEG-8000		6.33% Na2SO4	0.215M NaClO4	0.01 M NaPB	6.8	0.5 M TMAO	3.91	6.07	8.2	18		6.24	3.47	12.8	3.64	8.85	12.8	7.69	8.64					
S3	21	12.9% Dex-70	18.1% Ficoll-70			0.01 M NaPB	7.4							1.16		1.244	1.127	1.29	1.266	1.17	1.308					
S3	22	13.67% Dex-70	6.15% PEG-4000			0.01 M NaPB	7.4							1.37		1.378	1.122	1.69	1.583	1.48	1.65					
S3	23	20.0% Dex-70	13.50% PEG-1000			0.01 M NaPB	7.4							1.6		1.6	1.213	2	2.065	1.63	2.15					
S3	24	16.23% Dex-70	16.87% PEG-600			0.01 M NaPB	7.4							1.351		1.32	1.1	1.697	1.54	1.37	1.59					
S3	25	12.39% Dex-70	10.08% Ucon-4000			0.01 M NaPB	7.4							2.09		2.55	1.508	3.16	3.34	1.93	3.19					
S3	26	22.99% Ficoll-70	9.90% PEG-10000			0.01 M NaPB	7.4							1.434		1.263	1.059	1.7	1.46	1.46	1.572					
S3	27	24.67% Ficoll-70	10.42% PEG-8000			0.01 M NaPB	7.4							1.553		1.363	1.099	1.84	1.57	1.51	1.632					
S3	28	23.08% Ficoll-70	9.87% PEG-6000			0.01 M NaPB	7.4							1.361		1.195	1.021	1.61	1.342	1.36	1.461					
S3	29	19.12% Ficoll-70	15.47% Ucon-4000			0.01 M NaPB	7.4							2.29		2.67	1.63	3.55	3.65	2.22	3.14					
S3	30	15.00% PEG-8000	29.97% Ucon-4000			0.01 M NaPB	7.4							2.21		2.92	2.22	3.35	4.41	2.43	2.59					
S3	31	12.9% Dex-70	18.1% Ficoll-70		0.15M NaCl	0.01 M NaPB	7.4							1.16		1.164	1.155	1.21	1.253	1.03	1.19					
S3	32	13.67% Dex-70	6.15% PEG-4000		0.15M NaCl	0.01 M NaPB	7.4							1.41		1.34	1.106	1.663	1.623	1.48	1.69					
S3	33	20.0% Dex-70	13.50% PEG-1000		0.15M NaCl	0.01 M NaPB	7.4							1.77		1.67	1.31	2.38	2.037	1.77	2.15					
S3	34	16.23% Dex-70	16.87% PEG-600		0.15M NaCl	0.01 M NaPB	7.4							1.44		1.37	1.175	1.64	1.49	1.4	1.67					
S3	35	12.39% Dex-70	10.08% Ucon-4000		0.15M NaCl	0.01 M NaPB	7.4							2.245		2.51	1.61	3.23	3.52	2.4	3.37					
S3	36	22.99% Ficoll-70	9.90% PEG-10000		0.15M NaCl	0.01 M NaPB	7.4							1.44		1.298	1.108	1.71	1.613	1.55	1.75					
S3	37	24.67% Ficoll-70	10.42% PEG-8000		0.15M NaCl	0.01 M NaPB	7.4							1.54		1.402	1.133	1.74	1.694	1.6	1.9					
S3	38	23.08% Ficoll-70	9.87% PEG-6000		0.15M NaCl	0.01 M NaPB	7.4							1.33		1.17	1.07	1.63	1.478	1.4	1.57					
S3	39	19.12% Ficoll-70	15.47% Ucon-4000		0.15M NaCl	0.01 M NaPB	7.4							2.34		2.94	1.87	3.82	4.81	2.5	3.98					
S3	40	15.00% PEG-8000	29.97% Ucon-4000		0.15M NaCl	0.01 M NaPB	7.4							2.54		3.92	2.685	4.28	5.92	3.11	3.85					
S3	41	12.9% Dex-70	18.1% Ficoll-70		0.10M Na2SO4	0.01 M NaPB	7.4							1.18		1.358	1.21	1.41	1.463	1.36	1.475					
S3	42	13.67% Dex-70	6.15% PEG-4000		0.10M Na2SO4	0.01 M NaPB	7.4							1.649		1.684	1.242	2.004	2.015	1.46	1.94					
S3	43	20.0% Dex-70	13.50% PEG-1000		0.10M Na2SO4	0.01 M NaPB	7.4							1.85		2.11	1.347	2.438	2.78	2.71	2.87					
S3	44	16.23% Dex-70	16.87% PEG-600		0.10M Na2SO4	0.01 M NaPB	7.4							1.72		1.76	1.323	2.18	2.15	1.77	2.01					
S3	45	12.39% Dex-70	10.08% Ucon-4000		0.10M Na2SO4	0.01 M NaPB	7.4							2.85		3.6	2.03	5.14	6.54	2.32	5.11					
S3	46	22.99% Ficoll-70	9.90% PEG-10000		0.10M Na2SO4	0.01 M NaPB	7.4							1.72		1.549	1.178	2.15	1.8	1.69	2.05					
S3	47	24.67% Ficoll-70	10.42% PEG-8000		0.10M Na2SO4	0.01 M NaPB	7.4							1.79		1.686	1.25	2.316	2.1	1.54	2.168					
S3	48	23.08% Ficoll-70	9.87% PEG-6000		0.10M Na2SO4	0.01 M NaPB	7.4							1.643		1.499	1.15	1.931	1.84	1.65	1.79					
S3	49	19.12% Ficoll-70	15.47% Ucon-4000		0.10M Na2SO4	0.01 M NaPB	7.4							3.63		4.679	2.37	6.56	7.47	3.72	5.23					
S3	50	12.9% Dex-70	18.1% Ficoll-70		0.15M NaClO4	0.01 M NaPB	7.4							1.121		1.219	1.079	1.282	1.294	1.13	1.223					
S3	51	13.67% Dex-70	6.15% PEG-4000		0.15M NaClO4	0.01 M NaPB	7.4							1.48		1.54	1.214	1.81	1.828	1.46	1.758					
S3	52	20.0% Dex-70	13.50% PEG-1000		0.15M NaClO4	0.01 M NaPB	7.4							1.88		2.08	1.54	2.48	2.61	1.6	2.51					
S3	53	16.23% Dex-70	16.87% PEG-600		0.15M NaClO4	0.01 M NaPB	7.4							1.6		1.61	1.303	1.91	2.03	1.52	1.83					
S3	54	12.39% Dex-70	10.08% Ucon-4000		0.15M NaClO4	0.01 M NaPB	7.4							2.31		3.09	1.585	3.84	4.69	2.66	3.62					
S3	55	22.99% Ficoll-70	9.90% PEG-10000		0.15M NaClO4	0.01 M NaPB	7.4							1.557		1.465	1.138	1.952	1.79	1.6	1.84					
S3	56	24.67% Ficoll-70	10.42% PEG-8000		0.15M NaClO4	0.01 M NaPB	7.4							1.664		1.573	1.187	2.14	1.935	1.66	1.862					
S3	57	23.08% Ficoll-70	9.87% PEG-6000		0.15M NaClO4	0.01 M NaPB	7.4							1.512		1.38	1.151	1.889	1.66	1.48	1.7					
S3	58	19.12% Ficoll-70	15.47% Ucon-4000		0.15M NaClO4	0.01 M NaPB	7.4							2.49		3.24	1.771	4.21	5.02	2.65	3.72					
S3	59	15.00% PEG-8000	29.97% Ucon-4000		0.15M NaClO4	0.01 M NaPB	7.4							2.15		2.977	2.02	3.04	4.25	2.46	2.559					
S4	60	11.10% PEG-8000		6.33% Na2SO4		0.01 M NaPB	6.8		3.7	4.73	6	10.3	3.04	3.5	1.85	4.86	2.18	4.6	7.28	4.16	6.78					
S4	61	11.10% PEG-10000		6.33% Na2SO4		0.01 M NaPB	6.8		3.86	5.15	6.4	12.34	3.165	3.55	1.984	4.55	2.405	4.8	7.29	3.8	5.79					
S4	62	11.10% PEG-8000		6.33% Na2SO4		0.01 M NaPB	6.8	0.5 M sorbitol	6.21	8.72	11.1	21.2	4.45	5.36	2.48	8.23	3.48	8.84	13.48	6.67	11.11					
S4	63	11.10% PEG-8000		6.33% Na2SO4		0.01 M NaPB	6.8	0.5 M sucrose	6.3	9.8	13.2	28.7	4.55	6.11	2.49	8.46	3.75	10.43	14.21	7.7	11.38					
S4	64	11.10% PEG-8000		6.33% Na2SO4		0.01 M NaPB	6.8	0.5 M trehalose	6.88	10.7	15	32.1	4.66	5.69	2.44	7.97	3.59	9.86	14.9	6.87	11					
S4	65	11.10% PEG-8000		6.33% Na2SO4		0.01 M NaPB	6.8	0.5 M TMAO	5.9	8	10.7	20.2	4.11	3.86	2.45	6.27	2.87	5.7	9.06	4.71	6.91					
S5, S6	66	11.10% PEG-8000		6.33% Na2SO4	0.027M NaCl	0.01 M NaPB	6.8						3.106		1.864		2.167	4.857				3.2	0.97	0.7	0.57	2.661
S5, S6	67	11.10% PEG-8000		6.33% Na2SO4	0.053 M NaCl	0.01 M NaPB	6.8						3.272		1.905		2.236	5.229				2.95	0.95	0.57	0.52	2.789
S5, S6	68	11.10% PEG-8000		6.33% Na2SO4	0.107 M NaCl	0.01 M NaPB	6.8						3.49		1.969		2.348	5.701				2.8	0.88	0.6	0.46	2.879
S5, S6		11.10% PEG-8000		6.33% Na2SO4	0215M NaCl	0.01 M NaPB	6.8						3.761		2.107		2.471	6.5								3.122
S5, S6	69	11.10% PEG-8000		6.33% Na2SO4	0.543M NaCl	0.01 M NaPB	6.8						4.291		2.209		2.755	8.2				4.1	0.72	0.43	0.3	3.479
S5, S6		11.10% PEG-8000		6.33% Na2SO4	1.109 M NaCl	0.01 M NaPB	6.8						5.246		2.376		3.355	12								4.208
S5, S6		11.10% PEG-8000		6.33% Na2SO4	1.936 M NaCl	0.01 M NaPB	6.8						6.797		2.438		4.477	20								5.41
S5, S6	70	11.10% PEG-8000		6.33% Na2SO4	0.027M NaClO4	0.01 M NaPB	6.8						3.386		2.054		2.347	5.704				3.29	0.91	0.63	0.46	2.84
S5, S6	71	11.10% PEG-8000		6.33% Na2SO4	0.054M NaClO4	0.01 M NaPB	6.8						3.705		2.213		2.438	6.61				3.57	0.8	0.53	0.37	2.969
S5, S6	72	11.10% PEG-8000		6.33% Na2SO4	0.108M NaClO4	0.01 M NaPB	6.8						4.14		2.38		2.69	7.7				3.78	0.65	0.4	0.26	3.113
S5, S6		11.10% PEG-8000		6.33% Na2SO4	0.216 M NaClO4	0.01 M NaPB	6.8						4.584		2.578		2.888	9								3.208
S5, S6	73	11.10% PEG-8000		6.33% Na2SO4	0.549M NaClO4	0.01 M NaPB	6.8						5.141		2.471		3.092	11				4.1	0.335	0.19	0.109	3.122
S5, S6		11.10% PEG-8000		6.33% Na2SO4	1.157M NaClO4	0.01 M NaPB	6.8						5.759		2.04		3.423	17								2.662
S5, S6		11.10% PEG-8000		6.33% Na2SO4	1.759 M NaClO4	0.01 M NaPB	6.8						6.284		1.627		3.4	28								2.126
S5, S6	74	11.10% PEG-8000		6.33% Na2SO4	0.025 M NaSCN	0.01 M NaPB	6.8						3.469		1.996		2.301	5.576				3.27	0.9	0.637	0.468	2.762
S5, S6	75	11.10% PEG-8000		6.33% Na2SO4	0.050 M NaSCN	0.01 M NaPB	6.8						3.698		2.09		2.459	6.4				3.52	0.8	0.54	0.388	2.893
S5, S6	76	11.10% PEG-8000		6.33% Na2SO4	0.100 M NaSCN	0.01 M NaPB	6.8						3.983		2.265		2.589	7.4				3.7	0.678	0.431	0.29	3.074
S5, S6	77	11.10% PEG-8000		6.33% Na2SO4	0.215 M NaSCN	0.01 M NaPB	6.8						4.319		2.381		2.769	8				3.75	0.521	0.316	0.21	3.232
S5, S6	78	11.10% PEG-8000		6.33% Na2SO4	0.543 M NaSCN	0.01 M NaPB	6.8						4.566		2.218		2.868	8.694				3.51	0.405	0.253	0.164	2.9169
S5, S6		11.10% PEG-8000		6.33% Na2SO4	1.109M NaSCN	0.01 M NaPB	6.8						4.83		1.863		2.869	10.1								2.63
S5, S6		11.10% PEG-8000		6.33% Na2SO4	1.936M NaSCN	0.01 M NaPB	6.8						5.1		1.482		2.851	13.1								2.197
S5, S6	79	11.10% PEG-8000		6.33% Na2SO4	0.027 M NaH2PO4	0.01 M NaPB	6.8						5.086		1.888		2.255	5.2				2.9	1.28	0.89	0.64	2.562
S5, S6	80	11.10% PEG-8000		6.33% Na2SO4	0.054 M NaH2PO4	0.01 M NaPB	6.8						5.753		1.978		2.384	5.5				2.8	1.38	0.89	0.63	2.622
S5, S6	81	11.10% PEG-8000		6.33% Na2SO4	0.108 M NaH2PO4	0.01 M NaPB	6.8						6.7		2.125		2.542	6.2				2.61	1.41	0.86	0.57	2.631
S5, S6	82	11.10% PEG-8000		6.33% Na2SO4	0.217 M NaH2PO4	0.01 M NaPB	6.8						9.3		2.382		3.02	8.1				2.18	1.26	0.741	0.41	2.701
S5, S6	83	11.10% PEG-8000		6.33% Na2SO4	0.556 M NaH2PO4	0.01 M NaPB	6.8						20		3.222		4.583	15.3				1.5	0.88	0.406	0.18	2.529
S5, S6		11.10% PEG-8000		6.33% Na2SO4	1.160 M NaH2PO4	0.01 M NaPB	6.8						69		5.2		8.8	37								2.182
S5, S6		11.10% PEG-8000		6.33% Na2SO4	1.751 M NaH2PO4	0.01 M NaPB	6.8								8.7		18.8	78								2.135
S7	84	6.0% PEG-8000	12.0% Dex-75			0.01 M K/NaPB	7.4							1.409	1.154	1.49	1.232	1.7	1.77	1.469	1.709	1.22				1.128
S7	85	6.0% PEG-8000	12.0% Dex-75			0.01 M K/NaPB	7.4	0.5 M sorbitol						1.52	1.178	1.684	1.31	2.008	2.035	1.6	1.82	1.311				1.21
S7	86	6.0% PEG-8000	12.0% Dex-75			0.01 M K/NaPB	7.4	0.5 M sucrose						1.607	1.16	1.697	1.332	2.07	2.124	1.695	1.969	1.271				1.215
S7	87	6.0% PEG-8000	12.0% Dex-75			0.01 M K/NaPB	7.4	0.5 M trehalose						1.697	1.186	1.78	1.368	2.211	2.24	1.697	2.105	1.367				1.256
S7	88	6.0% PEG-8000	12.0% Dex-75			0.01 M K/NaPB	7.4	0.5 M TMAO						1.454	1.147	1.59	1.246	1.809	1.847	1.51	1.761	1.264				1.192

S8	89	12.94% Dex-70	18.06% Ficoll-70		0.15 M NaCl	0.01 M NaPB	7.4		1.16	1.208	1.25	1.35														
S8	90	13.03% Dex-70	5.86% PEG-10000		0.15 M NaCl	0.01 M NaPB	7.4		1.06	1.139	1.21	1.39														
S8	91	12.41% Dex-70	6.06% PEG-8000		0.15 M NaCl	0.01 M NaPB	7.4		1.04	1.13	1.209	1.416														
S8	92	13.48% Dex-70	6.00% PEG-6000		0.15 M NaCl	0.01 M NaPB	7.4		0.988	1.066	1.13	1.31														
S8	93	20.00% Dex-70	8.88% PEG-6000		0.15 M NaCl	0.01 M NaPB	7.4		1.08	1.246	1.41	1.9														
S8	94	13.67% Dex-70	6.15% PEG-4000		0.15 M NaCl	0.01 M NaPB	7.4		0.982	1.06	1.123	1.29														
S8	95	20.00% Dex-70	9.26% PEG-4000		0.15 M NaCl	0.01 M NaPB	7.4		1.084	1.288	1.463	1.945														
S8	96	17.26% Dex-70	12.13% PEG-1000		0.15 M NaCl	0.01 M NaPB	7.4		1.02	1.146	1.23	1.35														
S8	97	20.00% Dex-70	13.57% PEG-1000		0.15 M NaCl	0.01 M NaPB	7.4		1.107	1.26	1.394	1.791														
S8	98	16.23% Dex-70	16.87% PEG-600		0.15 M NaCl	0.01 M NaPB	7.4		1.032	1.106	1.171	1.33														
S8	99	12.39% Dex-70	10.08% Ucon-4000		0.15 M NaCl	0.01 M NaPB	7.4		1.36	1.68	2.04	3.13														
S8	100	10.00% Dex-70	8.00% Ucon-4000		0.15 M NaCl	0.01 M NaPB	7.4		1.235	1.56	1.7	2.6														
S8	101	22.99% Ficoll-70	9.90% PEG-10000		0.15 M NaCl	0.01 M NaPB	7.4		0.79	0.837	0.878	0.977														
S8	102	24.67% Ficoll-70	10.42% PEG-8000		0.15 M NaCl	0.01 M NaPB	7.4		0.747	0.829	0.876	0.99														
S8	103	23.08% Ficoll-70	9.87% PEG-6000		0.15 M NaCl	0.01 M NaPB	7.4		0.781	0.843	0.88	1.01														
S8	104	29.23% Ficoll-70	15.00% PEG-6000		0.15 M NaCl	0.01 M NaPB	7.4		0.86	1.002	1.097	1.42														
S8	105	13.01% Ficoll-70	9.93% Ucon-4000		0.15 M NaCl	0.01 M NaPB	7.4		1.18	1.4	1.61	2.18														
S8	106	19.12% Ficoll-70	15.47% Ucon-4000		0.15 M NaCl	0.01 M NaPB	7.4		1.59	2.1	2.65	4.52														
S8	107	15.00 PEG-8000	29.97% Ucon-4000		0.15 M NaCl	0.01 M NaPB	7.4		5	6.7	8.8	14.7														
S8	108	22.56% Ficoll-70	13.62% PEG-4000		0.15 M NaCl	0.01 M NaPB	7.4		0.788	0.866	0.914	1.055														
S9	109	17.30% PES	12.43% Dex-70		0.15 M NaCl	0.01 M NaPB	7.4							0.72	0.84		0.78	0.72								
S9	110	15.06% Ficoll-70	7.90% PEG-8000		0.15 M NaCl	0.01 M NaPB	7.4							1.17	1.01		1	1.21								
S9	111	17.31% PES	14.86% Ficoll-70		0.15 M NaCl	0.01 M NaPB	7.4							1.94	0.96		0.92	0.95								
S9	112	15.24% PES	6.96% PEG-8000		0.15 M NaCl	0.01 M NaPB	7.4							1.04	0.93		0.86	1.17								

Ref	# ATPS	Polymer-1	Polymer-2	Salt	salt additive	Buffer	pH	Osmolyte	logK DNP-Ala	logK DNP-n-val	logK DNP-n-leu	logK DNP-AO	logK 4-Aminophenol	logK Benzyl alcohol	logK Caffeine	logK Coumarin	logK Glucoside	logK Phenol	logK Methyl anthranilate	logK 2-Phenylethanol	logK Vanillin	logK Adenine	logK AMP	logK ADP	logK ATP	logK Adenosine
S1	1	11.10% PEG-8000		6.33% Na2SO4	0.215M NaCl	0.01 M NaPB	6.8		0.6464	0.7348	0.8543	1.144		0.6093	0.3243	0.6866	0.3927	0.8129	0.9818	0.6972	0.918					
S1	2	11.10% PEG-10000		6.33% Na2SO4	0.215M NaCl	0.01 M NaPB	6.8		0.601	0.7292	0.8376	1.1464		0.6176	0.3722	0.658	0.4648	0.8156	0.9814	0.7033	0.9196					
S1	3	11.10% PEG-8000		6.33% Na2SO4	0.215M NaCl	0.01 M NaPB	6.8	0.5 M sorbitol	0.7093	0.8854	1.0099	1.3729		0.7839	0.4346	0.9154	0.5974	1.0253	1.1703	0.8686	1.1149					
S1	4	11.10% PEG-8000		6.33% Na2SO4	0.215M NaCl	0.01 M NaPB	6.8	0.5 M sucrose	0.7067	0.896	1.0338	1.3986		0.8202	0.4065	0.9274	0.6075	1.0781	1.2154	0.9304	1.1212					
S1	5	11.10% PEG-8000		6.33% Na2SO4	0.215M NaCl	0.01 M NaPB	6.8	0.5 M TMAO	0.7324	0.8808	1.0208	1.3197		0.673	0.4501	0.7973	0.5328	0.8681	1.0708	0.7482	0.8797					
S2	6	6.0% PEG-8000	12.0 Dex-75		0.215M NaCl	0.01 M K/NaPB	7.4		-6.12E-03	0.0322	0.0535	0.1166		0.1895	0.0622	0.2071	0.0871	0.286	0.2853	0.2019	0.231					
S2	7	6.0% PEG-8000	12.0 Dex-75		0.215M NaCl	0.01 M K/NaPB	7.4	0.5 M sorbitol	0.0414	0.0888	0.1139	0.1923		0.2122	0.0704	0.241	0.1173	0.3255	0.3294	0.2502	0.2876					
S2	8	6.0% PEG-8000	12.0 Dex-75		0.215M NaCl	0.01 M K/NaPB	7.4	0.5 M sucrose	0.0584	0.0973	0.129	0.2106		0.236	0.0734	0.2497	0.1303	0.3491	0.346	0.2662	0.2982					
S2	9	6.0% PEG-8000	12.0 Dex-75		0.215M NaCl	0.01 M K/NaPB	7.4	0.5 M TMAO	0	0.0441	0.0682	0.1374		0.194	0.0821	0.2201	0.1004	0.2801	0.286	0.2219	0.2151					
S2	10	6.0% PEG-8000	12.0 Dex-75		0.215M NaCl	0.01 M K/NaPB	7.4	1.5 M TMAO	0.0453	0.1041	0.148	0.236		0.1915	0.1176	0.284	0.1335	0.3006	0.3922	0.2307	0.2596					
S2	11	6.0% PEG-8000	12.0 Dex-75		0.215M NaClO4	0.01 M K/NaPB	7.4		-0.0182	0.0124	0.0382	0.0966		0.2095	0.0781	0.2225	0.0966	0.2808	0.2978	0.2209	0.2183					
S2	12	6.0% PEG-8000	12.0 Dex-75		0.215M NaClO4	0.01 M K/NaPB	7.4	0.5 M sorbitol	-0.015	0.0269	0.0622	0.144		0.2472	0.1024	0.2953	0.1449	0.091	0.3897	0.2776	0.2951					
S2	13	6.0% PEG-8000	12.0 Dex-75		0.215M NaClO4	0.01 M K/NaPB	7.4	0.5 M sucrose	-0.0128	0.031	0.0682	0.152		0.2688	0.0955	0.296	0.1489	0.3795	0.4118	0.3086	0.3111					
S2	14	6.0% PEG-8000	12.0 Dex-75		0.215M NaClO4	0.01 M K/NaPB	7.4	0.5 M TMAO	-0.0155	0.0249	0.0577	0.1339		0.2003	0.0976	0.2507	0.1149	0.2876	0.3284	0.237	0.2017					
S2	15	6.0% PEG-8000	12.0 Dex-75		0.215M NaClO4	0.01 M K/NaPB	7.4	1.5 M TMAO	-2.61E-03	0.07	0.129	0.2681		0.2683	0.1559	0.3883	0.1804	0.3551	0.4912	0.3084	0.2499					
S2	16	11.10% PEG-8000		6.33% Na2SO4	0.215M NaClO4	0.01 M NaPB	6.8		0.5454	0.7152	0.8543	1.143		0.7839	0.412	1.031	0.4619	0.9965	1.2695	0.9133	1.0959					
S2	17	11.10% PEG-10000		6.33% Na2SO4	0.215M NaClO4	0.01 M NaPB	6.8		0.5647	0.7292	0.8597	1.1761		0.7634	0.4757	1.0128	0.5185	0.936	1.2014	0.8651	1.0934					
S2	18	11.10% PEG-8000		6.33% Na2SO4	0.215M NaClO4	0.01 M NaPB	6.8	0.5 M sorbitol	0.6042	0.7853	0.935	1.281		0.9031	0.5441	1.1818	0.618	1.0719	1.4698	0.9754	1.281					
S2	19	11.10% PEG-8000		6.33% Na2SO4	0.215M NaClO4	0.01 M NaPB	6.8	0.5 M sucrose	0.5422	0.7324	0.8882	1.2487		0.8062	0.4681	1.0821	0.5866	1.0945	1.3345	0.9854	1.1732					
S2	20	11.10% PEG-8000		6.33% Na2SO4	0.215M NaClO4	0.01 M NaPB	6.8	0.5 M TMAO	0.5922	0.7832	0.9138	1.2553		0.7952	0.5403	1.1072	0.5611	0.9469	1.1072	0.8859	0.9365					
S3	21	12.9% Dex-70	18.1% Ficoll-70			0.01 M NaPB	7.4							0.0645		0.0948	0.0519	0.1106	0.1024	0.0682	0.1166					
S3	22	13.67% Dex-70	6.15% PEG-4000			0.01 M NaPB	7.4							0.1367		0.1392	0.05	0.2279	0.1995	0.1703	0.2175					
S3	23	20.0% Dex-70	13.50% PEG-1000			0.01 M NaPB	7.4							0.2041		0.2041	0.0839	0.301	0.3149	0.2122	0.3324					
S3	24	16.23% Dex-70	16.87% PEG-600			0.01 M NaPB	7.4							0.1307		0.1206	0.0414	0.2297	0.1875	0.1367	0.2014					
S3	25	12.39% Dex-70	10.08% Ucon-4000			0.01 M NaPB	7.4							0.3201		0.4065	0.1784	0.4997	0.5237	0.2856	0.5038					
S3	26	22.99% Ficoll-70	9.90% PEG-10000			0.01 M NaPB	7.4							0.1565		0.1014	0.0249	0.2304	0.1644	0.1644	0.1965					
S3	27	24.67% Ficoll-70	10.42% PEG-8000			0.01 M NaPB	7.4							0.1912		0.1345	0.041	0.2648	0.1959	0.179	0.2127					
S3	28	23.08% Ficoll-70	9.87% PEG-6000			0.01 M NaPB	7.4							0.1339		0.0774	9.03E-03	0.2068	0.1278	0.1335	0.1647					
S3	29	19.12% Ficoll-70	15.47% Ucon-4000			0.01 M NaPB	7.4							0.3598		0.4265	0.2122	0.5502	0.5623	0.3464	0.4969					
S3	30	15.00% PEG-8000	29.97% Ucon-4000			0.01 M NaPB	7.4							0.3444		0.4654	0.3464	0.525	0.6444	0.3856	0.4133					
S3	31	12.9% Dex-70	18.1% Ficoll-70		0.15M NaCl	0.01 M NaPB	7.4							0.0645		0.066	0.0626	0.0828	0.098	0.0128	0.0755					
S3	32	13.67% Dex-70	6.15% PEG-4000		0.15M NaCl	0.01 M NaPB	7.4							0.1492		0.1271	0.0438	0.2209	0.2103	0.1703	0.2279					
S3	33	20.0% Dex-70	13.50% PEG-1000		0.15M NaCl	0.01 M NaPB	7.4							0.248		0.2227	0.1173	0.3766	0.309	0.248	0.3324					
S3	34	16.23% Dex-70	16.87% PEG-600		0.15M NaCl	0.01 M NaPB	7.4							0.1584		0.1367	0.07	0.2148	0.1732	0.1461	0.2227					
S3	35	12.39% Dex-70	10.08% Ucon-4000		0.15M NaCl	0.01 M NaPB	7.4							0.3512		0.3997	0.2068	0.5092	0.5465	0.3802	0.5276					
S3	36	22.99% Ficoll-70	9.90% PEG-10000		0.15M NaCl	0.01 M NaPB	7.4							0.1584		0.1133	0.0445	0.233	0.2076	0.1903	0.243					
S3	37	24.67% Ficoll-70	10.42% PEG-8000		0.15M NaCl	0.01 M NaPB	7.4							0.1875		0.1467	0.0542	0.2405	0.2289	0.2041	0.2788					
S3	38	23.08% Ficoll-70	9.87% PEG-6000		0.15M NaCl	0.01 M NaPB	7.4							0.1239		0.0682	0.0294	0.2122	0.1697	0.1461	0.1959					
S3	39	19.12% Ficoll-70	15.47% Ucon-4000		0.15M NaCl	0.01 M NaPB	7.4							0.3692		0.4683	0.2718	0.5821	0.6821	0.3979	0.5999					
S3	40	15.00% PEG-8000	29.97% Ucon-4000		0.15M NaCl	0.01 M NaPB	7.4							0.4048		0.5933	0.4289	0.6314	0.7723	0.4928	0.5855					
S3	41	12.9% Dex-70	18.1% Ficoll-70		0.10M Na2SO4	0.01 M NaPB	7.4							0.0719		0.1329	0.0828	0.1492	0.1652	0.1335	0.1688					
S3	42	13.67% Dex-70	6.15% PEG-4000		0.10M Na2SO4	0.01 M NaPB	7.4							0.2172		0.2263	0.0941	0.3019	0.3043	0.1644	0.2878					
S3	43	20.0% Dex-70	13.50% PEG-1000		0.10M Na2SO4	0.01 M NaPB	7.4							0.2672		0.3243	0.1294	0.387	0.444	0.433	0.4579					
S3	44	16.23% Dex-70	16.87% PEG-600		0.10M Na2SO4	0.01 M NaPB	7.4							0.2355		0.2455	0.1216	0.3385	0.3324	0.248	0.3032					
S3	45	12.39% Dex-70	10.08% Ucon-4000		0.10M Na2SO4	0.01 M NaPB	7.4							0.4548		0.5563	0.3075	0.711	0.8156	0.3655	0.7084					
S3	46	22.99% Ficoll-70	9.90% PEG-10000		0.10M Na2SO4	0.01 M NaPB	7.4							0.2355		0.1901	0.0711	0.3324	0.2553	0.2279	0.3118					
S3	47	24.67% Ficoll-70	10.42% PEG-8000		0.10M Na2SO4	0.01 M NaPB	7.4							0.2529		0.2269	0.0969	0.3647	0.3222	0.1875	0.3361					
S3	48	23.08% Ficoll-70	9.87% PEG-6000		0.10M Na2SO4	0.01 M NaPB	7.4							0.2156		0.1758	0.0607	0.2858	0.2648	0.2175	0.2529					
S3	49	19.12% Ficoll-70	15.47% Ucon-4000		0.10M Na2SO4	0.01 M NaPB	7.4							0.5599		0.6702	0.3747	0.8169	0.8733	0.5705	0.7185					
S3	50	12.9% Dex-70	18.1% Ficoll-70		0.15M NaClO4	0.01 M NaPB	7.4							0.0496		0.086	0.033	0.1079	0.1119	0.0531	0.0874					
S3	51	13.67% Dex-70	6.15% PEG-4000		0.15M NaClO4	0.01 M NaPB	7.4							0.1703		0.1875	0.0842	0.2577	0.262	0.1644	0.245					
S3	52	20.0% Dex-70	13.50% PEG-1000		0.15M NaClO4	0.01 M NaPB	7.4							0.2742		0.3181	0.1875	0.3945	0.4166	0.2041	0.3997					
S3	53	16.23% Dex-70	16.87% PEG-600		0.15M NaClO4	0.01 M NaPB	7.4							0.2041		0.2068	0.1149	0.281	0.3075	0.1818	0.2625					
S3	54	12.39% Dex-70	10.08% Ucon-4000		0.15M NaClO4	0.01 M NaPB	7.4							0.3636		0.49	0.2	0.5843	0.6712	0.4249	0.5587					
S3	55	22.99% Ficoll-70	9.90% PEG-10000		0.15M NaClO4	0.01 M NaPB	7.4							0.1923		0.1658	0.0561	0.2905	0.2529	0.2041	0.2648					
S3	56	24.67% Ficoll-70	10.42% PEG-8000		0.15M NaClO4	0.01 M NaPB	7.4							0.2212		0.1967	0.0745	0.3304	0.2867	0.2201	0.27					
S3	57	23.08% Ficoll-70	9.87% PEG-6000		0.15M NaClO4	0.01 M NaPB	7.4							0.1796		0.1399	0.0611	0.2762	0.2201	0.1703	0.2304					
S3	58	19.12% Ficoll-70	15.47% Ucon-4000		0.15M NaClO4	0.01 M NaPB	7.4							0.3962		0.5105	0.2482	0.6243	0.7007	0.4232	0.5705					
S3	59	15.00% PEG-8000	29.97% Ucon-4000		0.15M NaClO4	0.01 M NaPB	7.4							0.3324		0.4738	0.3054	0.4829	0.6284	0.3909	0.4081					
S4	60	11.10% PEG-8000		6.33% Na2SO4		0.01 M NaPB	6.8		0.5682	0.6749	0.7782	1.0128	0.4829	0.5441	0.2672	0.6866	0.3385	0.6628	0.8621	0.6191	0.8312					
S4	61	11.10% PEG-10000		6.33% Na2SO4		0.01 M NaPB	6.8		0.5866	0.7118	0.8062	1.0913	0.5004	0.5502	0.2975	0.658	0.3811	0.6812	0.8627	0.5798	0.7627					
S4	62	11.10% PEG-8000		6.33% Na2SO4		0.01 M NaPB	6.8	0.5 M sorbitol	0.7931	0.9405	1.0453	1.3263	0.6484	0.7292	0.3945	0.9154	0.5416	0.9465	1.1297	0.8241	1.0457					
S4	63	11.10% PEG-8000		6.33% Na2SO4		0.01 M NaPB	6.8	0.5 M sucrose	0.7993	0.9912	1.1206	1.4579	0.658	0.786	0.3962	0.9274	0.574	1.0183	1.1526	0.8865	1.0561					
S4	64	11.10% PEG-8000		6.33% Na2SO4		0.01 M NaPB	6.8	0.5 M trehalose	0.8376	1.0294	1.1761	1.5065	0.6684	0.7551	0.3874	0.9015	0.5551	0.9939	1.1732	0.837	1.0414					
S4	65	11.10% PEG-8000		6.33% Na2SO4		0.01 M NaPB	6.8	0.5 M TMAO	0.7709	0.9031	1.0294	1.3054	0.6138	0.5866	0.3892	0.7973	0.4579	0.7559	0.9571	0.673	0.8395					
S5, S6	66	11.10% PEG-8000		6.33% Na2SO4	0.027M NaCl	0.01 M NaPB	6.8						0.4922		0.2704		0.3359	0.6864				0.5051	-0.0132	-0.1549	-0.2441	0.425
S5, S6	67	11.10% PEG-8000		6.33% Na2SO4	0.053 M NaCl	0.01 M NaPB	6.8						0.5148		0.2799		0.3495	0.7184				0.4698	-0.0223	-0.2441	-0.284	0.4454
S5, S6	68	11.10% PEG-8000		6.33% Na2SO4	0.107 M NaCl	0.01 M NaPB	6.8						0.5428		0.2942		0.3707	0.756				0.4472	-0.0555	-0.2218	-0.3372	0.4592
S5, S6		11.10% PEG-8000		6.33% Na2SO4	0215M NaCl	0.01 M NaPB	6.8						0.5753		0.3237		0.3929	0.8129								0.4944
S5, S6	69	11.10% PEG-8000		6.33% Na2SO4	0.543M NaCl	0.01 M NaPB	6.8						0.6326		0.3442		0.4401	0.9138				0.6128	-0.1427	-0.3665	-0.5229	0.5415
S5, S6		11.10% PEG-8000		6.33% Na2SO4	1.109 M NaCl	0.01 M NaPB	6.8						0.7198		0.3758		0.5257	1.0792								0.6241
S5, S6		11.10% PEG-8000		6.33% Na2SO4	1.936 M NaCl	0.01 M NaPB	6.8						0.8323		0.387		0.651	1.301								0.7332

S5, S6	70	11.10% PEG-8000		6.33% Na2SO4	0.027M NaClO4	0.01 M NaPB	6.8						0.5297		0.3126		0.3705	0.7562				0.5172	-0.041	-0.2007	-0.3372	0.4533
S5, S6	71	11.10% PEG-8000		6.33% Na2SO4	0.054M NaClO4	0.01 M NaPB	6.8						0.5688		0.345		0.387	0.8202				0.5527	-0.0969	-0.2757	-0.4318	0.4726
S5, S6	72	11.10% PEG-8000		6.33% Na2SO4	0.108M NaClO4	0.01 M NaPB	6.8						0.617		0.3766		0.4298	0.8865				0.5775	-0.1871	-0.3979	-0.585	0.4932
S5, S6		11.10% PEG-8000		6.33% Na2SO4	0.216 M NaClO4	0.01 M NaPB	6.8						0.6612		0.4113		0.4606	0.9542								0.5062
S5, S6	73	11.10% PEG-8000		6.33% Na2SO4	0.549M NaClO4	0.01 M NaPB	6.8						0.711		0.3929		0.4902	1.0414				0.6128	-0.475	-0.7212	-0.9626	0.4944
S5, S6		11.10% PEG-8000		6.33% Na2SO4	1.157M NaClO4	0.01 M NaPB	6.8						0.7603		0.3096		0.5344	1.2304								0.4252
S5, S6		11.10% PEG-8000		6.33% Na2SO4	1.759 M NaClO4	0.01 M NaPB	6.8						0.7982		0.2114		0.5315	1.4472								0.3276
S5, S6	74	11.10% PEG-8000		6.33% Na2SO4	0.025 M NaSCN	0.01 M NaPB	6.8						0.5402		0.3002		0.3619	0.7463				0.5145	-0.0458	-0.1959	-0.3298	0.4412
S5, S6	75	11.10% PEG-8000		6.33% Na2SO4	0.050 M NaSCN	0.01 M NaPB	6.8						0.568		0.3201		0.3908	0.8062				0.5465	-0.0969	-0.2676	-0.4112	0.4613
S5, S6	76	11.10% PEG-8000		6.33% Na2SO4	0.100 M NaSCN	0.01 M NaPB	6.8						0.6002		0.3551		0.4131	0.8692				0.5682	-0.1688	-0.3655	-0.5376	0.4877
S5, S6	77	11.10% PEG-8000		6.33% Na2SO4	0.215 M NaSCN	0.01 M NaPB	6.8						0.6354		0.3768		0.4423	0.9031				0.574	-0.2832	-0.5003	-0.6778	0.5095
S5, S6	78	11.10% PEG-8000		6.33% Na2SO4	0.543 M NaSCN	0.01 M NaPB	6.8						0.6595		0.346		0.4576	0.9392				0.5453	-0.3925	-0.5969	-0.7852	0.4649
S5, S6		11.10% PEG-8000		6.33% Na2SO4	1.109M NaSCN	0.01 M NaPB	6.8						0.6839		0.2702		0.4577	1.0043								0.42
S5, S6		11.10% PEG-8000		6.33% Na2SO4	1.936M NaSCN	0.01 M NaPB	6.8						0.7076		0.1708		0.455	1.1173								0.3418
S5, S6	79	11.10% PEG-8000		6.33% Na2SO4	0.027 M NaH2PO4	0.01 M NaPB	6.8						0.7064		0.276		0.3531	0.716				0.4624	0.1072	-0.0506	-0.1938	0.4086
S5, S6	80	11.10% PEG-8000		6.33% Na2SO4	0.054 M NaH2PO4	0.01 M NaPB	6.8						0.7599		0.2962		0.3773	0.7404				0.4472	0.1399	-0.0506	-0.2007	0.4186
S5, S6	81	11.10% PEG-8000		6.33% Na2SO4	0.108 M NaH2PO4	0.01 M NaPB	6.8						0.8261		0.3274		0.4052	0.7924				0.4166	0.1492	-0.0655	-0.2441	0.4201
S5, S6	82	11.10% PEG-8000		6.33% Na2SO4	0.217 M NaH2PO4	0.01 M NaPB	6.8						0.9685		0.3769		0.48	0.9085				0.3385	0.1004	-0.1302	-0.3872	0.4315
S5, S6	83	11.10% PEG-8000		6.33% Na2SO4	0.556 M NaH2PO4	0.01 M NaPB	6.8						1.301		0.5081		0.6611	1.1847				0.1761	-0.0555	-0.3915	-0.7447	0.4029
S5, S6		11.10% PEG-8000		6.33% Na2SO4	1.160 M NaH2PO4	0.01 M NaPB	6.8						1.8388		0.716		0.9445	1.5682								0.3389
S5, S6		11.10% PEG-8000		6.33% Na2SO4	1.751 M NaH2PO4	0.01 M NaPB	6.8								0.9395		1.2742	1.8921								0.3294
S7	84	6.0% PEG-8000	12.0% Dex-75			0.01 M K/NaPB	7.4							0.1489	0.0622	0.1732	0.0906	0.2304	0.248	0.167	0.2327	0.0864				0.0523
S7	85	6.0% PEG-8000	12.0% Dex-75			0.01 M K/NaPB	7.4	0.5 M sorbitol						0.1818	0.0711	0.2263	0.1173	0.3028	0.3086	0.2041	0.2601	0.1176				0.0828
S7	86	6.0% PEG-8000	12.0% Dex-75			0.01 M K/NaPB	7.4	0.5 M sucrose						0.206	0.0645	0.2297	0.1245	0.316	0.3272	0.2292	0.2942	0.1041				0.0846
S7	87	6.0% PEG-8000	12.0% Dex-75			0.01 M K/NaPB	7.4	0.5 M trehalose						0.2297	0.0741	0.2504	0.1361	0.3446	0.3502	0.2297	0.3233	0.1358				0.099
S7	88	6.0% PEG-8000	12.0% Dex-75			0.01 M K/NaPB	7.4	0.5 M TMAO						0.1626	0.0596	0.2014	0.0955	0.2574	0.2665	0.179	0.2458	0.1017				0.0763
S8	89	12.94% Dex-70	18.06% Ficoll-70		0.15 M NaCl	0.01 M NaPB	7.4		0.0645	0.0821	0.0969	0.1303														
S8	90	13.03% Dex-70	5.86% PEG-10000		0.15 M NaCl	0.01 M NaPB	7.4		0.0253	0.0565	0.0828	0.143														
S8	91	12.41% Dex-70	6.06% PEG-8000		0.15 M NaCl	0.01 M NaPB	7.4		0.017	0.0531	0.0824	0.1511														
S8	92	13.48% Dex-70	6.00% PEG-6000		0.15 M NaCl	0.01 M NaPB	7.4		-5.24E-03	0.0278	0.0531	0.1173														
S8	93	20.00% Dex-70	8.88% PEG-6000		0.15 M NaCl	0.01 M NaPB	7.4		0.0334	0.0955	0.1492	0.2788														
S8	94	13.67% Dex-70	6.15% PEG-4000		0.15 M NaCl	0.01 M NaPB	7.4		-7.89E-03	0.0253	0.0504	0.1106														
S8	95	20.00% Dex-70	9.26% PEG-4000		0.15 M NaCl	0.01 M NaPB	7.4		0.035	0.1099	0.1652	0.2889														
S8	96	17.26% Dex-70	12.13% PEG-1000		0.15 M NaCl	0.01 M NaPB	7.4		8.60E-03	0.0592	0.0899	0.1303														
S8	97	20.00% Dex-70	13.57% PEG-1000		0.15 M NaCl	0.01 M NaPB	7.4		0.0441	0.1004	0.1443	0.2531														
S8	98	16.23% Dex-70	16.87% PEG-600		0.15 M NaCl	0.01 M NaPB	7.4		0.0137	0.0438	0.0686	0.1239														
S8	99	12.39% Dex-70	10.08% Ucon-4000		0.15 M NaCl	0.01 M NaPB	7.4		0.1335	0.2253	0.3096	0.4955														
S8	100	10.00% Dex-70	8.00% Ucon-4000		0.15 M NaCl	0.01 M NaPB	7.4		0.0917	0.1931	0.2304	0.415														
S8	101	22.99% Ficoll-70	9.90% PEG-10000		0.15 M NaCl	0.01 M NaPB	7.4		-0.1024	-0.0773	-0.0565	-0.0101														
S8	102	24.67% Ficoll-70	10.42% PEG-8000		0.15 M NaCl	0.01 M NaPB	7.4		-0.1267	-0.0814	-0.0575	-4.36E-03														
S8	103	23.08% Ficoll-70	9.87% PEG-6000		0.15 M NaCl	0.01 M NaPB	7.4		-0.1073	-0.0742	-0.0555	4.32E-03														
S8	104	29.23% Ficoll-70	15.00% PEG-6000		0.15 M NaCl	0.01 M NaPB	7.4		-0.0655	8.68E-04	0.0402	0.1523														
S8	105	13.01% Ficoll-70	9.93% Ucon-4000		0.15 M NaCl	0.01 M NaPB	7.4		0.0719	0.1461	0.2068	0.3385														
S8	106	19.12% Ficoll-70	15.47% Ucon-4000		0.15 M NaCl	0.01 M NaPB	7.4		0.2014	0.3222	0.4232	0.6551														
S8	107	15.00 PEG-8000	29.97% Ucon-4000		0.15 M NaCl	0.01 M NaPB	7.4		0.699	0.8261	0.9445	1.1673														
S8	108	22.56% Ficoll-70	13.62% PEG-4000		0.15 M NaCl	0.01 M NaPB	7.4		-0.1035	-0.0625	-0.0391	0.0233														
S9	109	17.30% PES	12.43% Dex-70		0.15 M NaCl	0.01 M NaPB	7.4							-0.1427	-0.0757		-0.1079	-0.1427								
S9	110	15.06% Ficoll-70	7.90% PEG-8000		0.15 M NaCl	0.01 M NaPB	7.4							0.0682	4.32E-03		0	0.0828								
S9	111	17.31% PES	14.86% Ficoll-70		0.15 M NaCl	0.01 M NaPB	7.4							0.2878	-0.0177		-0.0362	-0.0223								
S9	112	15.24% PES	6.96% PEG-8000		0.15 M NaCl	0.01 M NaPB	7.4							0.017	-0.0315		-0.0655	0.0682								

Table S2. Partition coefficients and logarithms of partition coefficients for various proteins in aqueous two-phase systems of various compositions (NaPB – sodium phosphate buffer, K/NaPB – sodium/potassium phosphate buffer)

Ref	# ATPS	Polymer-1	Polymer-2	Salt	salt additive	Buffer	pH	Additive	K HSA	K a-Chymotrypsin	K a-Chymotrypsinogen A	K Con A	K Cyt c	K Hemoglobin bovine	K Hemoglobin human	K bLGA	K bLGB	K Lysozyme	K Rnase A	K Rnase B	K Subtilisin A	K Trypsinogen	K Lipase
S4	1	11.10% PEG-8000		6.33% Na2SO4		0.01 M NaPB	6.8			0.117	0.429	0.192						0.406					0.618
S4	2	11.10% PEG-10000		6.33% Na2SO4		0.01 M NaPB	6.8			0.0924	0.379	0.195						0.411					0.64
S4	3	11.10% PEG-8000		6.33% Na2SO4		0.01 M NaPB	6.8	0.5 M sorbitol		0.0576	0.204	0.159						0.145					0.569
S4	4	11.10% PEG-8000		6.33% Na2SO4		0.01 M NaPB	6.8	0.5 M sucrose		0.047	0.157	0.144						0.055					0.537
S4	5	11.10% PEG-8000		6.33% Na2SO4		0.01 M NaPB	6.8	0.5 M trehalose		0.0271	0.119	0.116						0.045					0.516
S4	6	11.10% PEG-8000		6.33% Na2SO4		0.01 M NaPB	6.8	0.5 M TMAO		0.112	0.456	0.182						0.297					0.595
S2	7	6.0% PEG-8000	12.0 Dex-75		0.215M NaCl	0.01 M K/NaPB	7.4			0.927	2.75	1.481						2.38					0.801
S2	8	6.0% PEG-8000	12.0 Dex-75		0.215M NaCl	0.01 M K/NaPB	7.4	0.5 M sorbitol		4.148	1.177	1.17						3.584					0.801
S2	9	6.0% PEG-8000	12.0 Dex-75		0.215M NaCl	0.01 M K/NaPB	7.4	0.5 M sucrose		5.184	1.072	1.156						3.766					0.817
S2	10	6.0% PEG-8000	12.0 Dex-75		0.215M NaCl	0.01 M K/NaPB	7.4	0.5 M TMAO		0.967	3.1	1.539						2.455					0.83
S2	11	6.0% PEG-8000	12.0 Dex-75		0.215M NaCl	0.01 M K/NaPB	7.4	1.5 M TMAO		1.56	4.45	0.928						3.848					0.757
S2	12	6.0% PEG-8000	12.0 Dex-75		0.215M NaClO4	0.01 M K/NaPB	7.4			1.22	3.646	0.213						20.3					0.727
S2	13	6.0% PEG-8000	12.0 Dex-75		0.215M NaClO4	0.01 M K/NaPB	7.4	0.5 M sorbitol		1.634	6.519	0.246						46.4					0.791
S2	14	6.0% PEG-8000	12.0 Dex-75		0.215M NaClO4	0.01 M K/NaPB	7.4	0.5 M sucrose		1.845	6.245	0.267						49.1					0.849
S2	15	6.0% PEG-8000	12.0 Dex-75		0.215M NaClO4	0.01 M K/NaPB	7.4	0.5 M TMAO		1.4	4.55	0.227						22.3					0.739
S2	16	6.0% PEG-8000	12.0 Dex-75		0.215M NaClO4	0.01 M K/NaPB	7.4	1.5 M TMAO		1.603	7.451	0.235						38.9					0.67
S10	17	12.41 Dex-75	6.06% PEG-8000			0.01 M NaPB	7.4				2.71		0.29	0.074	0.131			2.36	0.489	0.455		0.89	0.716
S10	18	10.75 Dex-75	5.22% PEG-8000			0.01 M NaPB												1.68	0.559				0.733
S10	19	8.99 Dex-75	4.34% PEG-8000			0.01 M NaPB												1.46	0.686				0.773
S10	20	12.39 Dex-75	10.08% Ucon-4000			0.01 M NaPB	7.4				1.78		0.12					2.95	0.247	0.265		0.702	0.658
S10	21	10.42 Dex-75	8.41% Ucon-4000			0.01 M NaPB	7.4							0.053	0.117			2.69	0.355				0.649
S10	22	8.23 Dex-75	6.82% Ucon-4000			0.01 M NaPB	7.4											2.2	0.537				0.761
S11	23	13.95% PEG-8000		8.06% Na2SO4		0.01 M UB	7.4			0.097	0.243	0.072			0.082	0.008	0.061	0.236	0.027	0.099	1.124	0.038	
S11	24	13.95% PEG-8000		8.06% Na2SO4	0.15 M NaCl	0.01 M UB	7.4			0.101	0.324	0.066			0.113	0.011	0.043	0.57	0.023	0.083	1.502	0.036	
S11	25	13.95% PEG-8000		8.06% Na2SO4	0.33 M NaCl	0.01 M UB	7.4			0.121	0.778	0.069			0.109	0.015	0.048	3.969	0.031	0.05	1.862	0.071	
S11	26	13.95% PEG-8000		8.06% Na2SO4	0.15M NaSCN	0.01 M UB	7.4			0.093	0.138	0.071		0.041	0.115	0.011	0.051	13.99	0.04	0.099	1.873	0.024	
S11	27	13.95% PEG-8000		8.06% Na2SO4	0.40M NaSCN	0.01 M UB	7.4			0.157	0.801	0.088		0.05	0.174	0.013	0.068	55.4	0.071	0.106	2.217	0.073	
S11	28	15.00% PEG-8000				1.4M K/NaPB	7.4		0.353	0.306		0.14		0.246	0.489	0.131	0.141		0.104	0.069	2.088	0.121	
S11	29	15.70% PEG-600		9.47% Na2SO4	0.15 M NaCl	0.058M K/NaPB	7.4		0.38	3.055	5.73	0.337	0.152	2.253	5.043	0.167	0.248	6.633	1.797	0.847	1.975	4.061	
S11	30	15.70% PEG-600		9.47% Na2SO4		0.085M K/NaPB	7.4		1.111	2.849	5.18	0.393	0.093	2.52	6.67	0.322	0.399	5.512	1.842	0.953	1.984	3.173	
S11	31	15.70% PEG-600		9.47% Na2SO4	0.15 M NaCl	0.085M K/NaPB	7.4		0.424	4.228	11.54	0.275	0.049	3.214	8.995	0.161	0.236	10.04	2.359	0.88	1.948	6.813	
S11	32	15.70% PEG-600		9.47% Na2SO4	0.40M NaSCN	0.085M K/NaPB	7.4			8.515	33.73	0.267	0.02	5.717	11.85	0.061	0.147	81.3	5.018	1.065	2.229	16.5	
S11	33	15.70% PEG-600		9.47% Na2SO4		0.17M K/NaPB	7.4		1.982	5.106	17.61	0.419	0.026	5.152	8.59	0.259	0.342	12.04	2.702	1.051	2.348	8.585	
S11	34	15.70% PEG-600		9.47% Na2SO4	0.15 M NaCl	0.17M K/NaPB	7.4		0.639	7.381	44.23	0.454	0.0083	5.889		0.144	0.275	23.22	3.44	1.108	2.293	15.14	
S11	35	15.70% PEG-600		9.47% Na2SO4	0.30 M NaCl	0.17M K/NaPB	7.4		0.364	11.55	76.63	0.591	0.029	5.77		0.124	0.199	85.6	6.299	1.274	2.419	28.34	
S11	36	15.70% PEG-600		9.47% Na2SO4	0.40 M NaSCN	0.17M K/NaPB	7.4			14.41	28.43		0.07	9.051		0.061	0.149	93.7	7.351	1.324	2.426	77.5	
S11	37	15.30% PEG-600		9.46% Na2SO4	0.15 M NaCl	0.15M K/NaPB	7.4		2.595	6.451	31.58	0.328	0.037	4.267	12.03	0.254	0.32	6.737	2.931	0.947	1.071	15.87	
S11	38	13.75% PEG-600				1.55M K/NaPB	7.4			28.1			0.035	14.71		46.5	5.12	65.3	29.89	2.073	1.597		
S12	39	9.00% PEG-8000	19.0% Ficoll-70			0.01 M K/NaPB	7.4			0.18	0.519	0.263				0.125	0.109	0.186	0.238	0.195			
S12	40	9.00% PEG-8000	19.0% Ficoll-70			0.01 M K/NaPB	7.4	0.5 M TMAO		0.201	0.687	0.258				0.166	0.121	0.171	0.244	0.19			
S12	41	9.00% PEG-8000	19.0% Ficoll-70			0.01 M K/NaPB	7.4	1.5 M TMAO		0.263	1.367	0.263				0.214	0.17	0.179	0.235	0.159			
S12	42	9.00% PEG-8000	19.0% Ficoll-70			0.01 M K/NaPB	7.4	1.5 M TMAO		0.333	3.06	0.267				0.42	0.312	0.162	0.241	0.142			
S12	43	11.90% Dex-70	15.7% PEG-600		0.15 M Na2SO4	0.01 M K/NaPB	7.4		0.518	1.475	2.491	0.235				0.365	0.332	0.765	0.885	1.078			
S12	44	11.90% Dex-70	15.7% PEG-600		0.15 M Na2SO4	0.01 M K/NaPB	7.4	0.5 M TMAO	0.629	1.784	3.296	0.229				0.368	0.367	0.79	0.959	1.187			
S12	45	11.90% Dex-70	15.7% PEG-600		0.15 M Na2SO4	0.01 M K/NaPB	7.4	1.5 M TMAO	0.887	2.306	3.781	0.24				0.461	0.505	0.9	1.061	1.29			
S12	46	11.90% Dex-70	15.7% PEG-600		0.15 M Na2SO4	0.01 M K/NaPB	7.4	1.5 M TMAO	1.717	3.185	5.614	0.241				0.791	0.75	1.03	1.283	1.531			
S12	47	11.90% Dex-70	15.7% PEG-600		0.15 M Na2SO4	0.01 M K/NaPB	7.4	1.95 M TMAO	3.909	3.978	7.575	0.252				1.282	1.298	1.16	1.506	1.736			
S13	48	12.4% Dex-70	6.1% PEG-8000		0.15 M NaCl	0.01 M NaPB	7.4				2.71		0.29	0.074	0.131			2.36	0.489	0.455		0.89	0.716
S13	49	12.4% Dex-70	10.1% Ucon-4000		0.15 M NaCl	0.01 M NaPB	7.4				1.78		0.12	0.053	0.117			2.95	0.247	0.265		0.702	0.658
S13	50	15.0% PEG-8000	30.0% Ucon-4000		0.15 M NaCl	0.01 M NaPB	7.4				0.0098							0.036	0.014			0.015	0.358
S13	51	15.10% Ficoll-70	7.90% PEG-8000		0.15 M NaCl	0.01 M NaPB	7.4				1.04			0.094	0.153			0.91	0.466	0.44		0.58	0.733
S13	52	13.00% Ficoll-70	9.93% Ucon-4000		0.15 M NaCl	0.01 M NaPB	7.4				0.638			0.052	0.074			1	0.25	0.237		0.345	0.634
S13	53	15.2% PES-100	6.96% PEG-8000		0.15 M NaCl	0.01 M NaPB	7.4				2.98			0.148	0.22			1.29	0.604	0.703		0.967	0.75
S13	54	12.9% PES-100	7.68% Ucon-4000		0.15 M NaCl	0.01 M NaPB	7.4				1.8			0.208	0.282			1.28	0.506	0.63		0.779	0.78
S1	55	11.10% PEG-8000		6.33% Na2SO4	0.215M NaCl	0.01 M NaPB	6.8			0.11	0.543	0.183						0.983					0.584
S1	56	11.10% PEG-10000		6.33% Na2SO4	0.215M NaCl	0.01 M NaPB	6.8			0.0814	0.472	0.173						0.929					0.585
S1	57	11.10% PEG-8000		6.33% Na2SO4	0.215M NaCl	0.01 M NaPB	6.8	0.5 M sorbitol		0.0412	0.335	0.146						0.531					0.534
S1	58	11.10% PEG-8000		6.33% Na2SO4	0.215M NaCl	0.01 M NaPB	6.8	0.5 M sucrose		0.043	0.365	0.13						0.335					0.522
S1	59	11.10% PEG-8000		6.33% Na2SO4	0.215M NaCl	0.01 M NaPB	6.8	0.5 M TMAO		0.0816	0.601	0.167						0.786					0.538
S14	60	12.33% Dex-69	6.05% PEG-8000			0.01 M NaPB	7.4		0.076	0.58	1.164	0.125	0.067	0.158	0.245	0.375	0.386	0.225	0.225	0.555	1.137	0.422	
S14	61	12.33% Dex-69	6.05% PEG-8000		0.15 M NaCl	0.01 M NaPB	7.4			0.859	2.42	0.133	0.212	0.136	0.207	0.072	0.138	1.939	0.583	0.589	1.469	0.812	
S14	62	12.33% Dex-69	6.05% PEG-8000		1.05 M NaCl	0.01 M NaPB	7.4			1.346	6.71	0.151	0.23	0.267	0.355	0.054	0.256	10.4	0.757	0.644	2.023	1.553	
S14	63	12.33% Dex-69	6.05% PEG-8000		0.083M Na2SO4	0.01 M NaPB	7.4		0.025	0.649	1.643	0.148	0.054	0.146	0.182	0.094	0.162	0.451	0.323	0.386	1.318	0.491	
S14	64	12.33% Dex-69	6.05% PEG-8000		0.30 M Na2SO4	0.01 M NaPB	7.4			0.594	2.277	0.141	0.04	0.123	0.166	0.05	0.123	0.688	0.401	0.317	1.575	0.579	
S14	65	12.33% Dex-69	6.05% PEG-8000		0.17 M CsCl	0.01 M NaPB	7.4			0.785	2.432	0.133	0.199	0.117	0.179	0.064	0.136	2.392	0.553	0.626	1.225	0.702	
S14	66	12.33% Dex-69	6.05% PEG-8000		0.80 M CsCl	0.01 M NaPB	7.4			1.118	5.4	0.141	0.197	0.176	0.262	0.053	0.228	7.49	0.655	0.536	1.659	0.884	
S14	67	12.33% Dex-69	6.05% PEG-8000		0.094 M NaClO4	0.01 M NaPB	7.4			0.93	3.653	0.143	0.347	0.144	0.179	0.056	0.182	14.6	0.748	0.544	1.353	1.028	
S14	68	12.33% Dex-69	6.05% PEG-8000		0.43 M NaClO4	0.01 M NaPB	7.4			1.069	5.24	0.151	0.37	0.192	0.264	0.052	0.121	53.3	0.771	0.579	1.627	1.003	
S14	69	12.33% Dex-69	6.05% PEG-8000		0.17 M NaSCN	0.01 M NaPB	7.4			0.995	3.87	0.145	0.36	0.134	0.242	0.054	0.118	10	0.799	0.579	1.483	0.987	
S14	70	12.33% Dex-69	6.05% PEG-8000		1.26 M NaSCN	0.01 M NaPB	7.4			1.033	4.17	0.171	0.249	0.311	0.518	0.064	0.208	23	0.726	0.658	1.559	0.775	
S15	71	12.0% Dex-75	6.0% PEG-8000			0.01 M K/NaPB	7.4			0.42	1	0.236			0.126	0.46	0.33	0.23	0.313	0.781		0.357	
S15	72	12.0% Dex-75	6.0% PEG-8000			0.01 M K/NaPB	7.4	0.5 M sorbitol		0.427	1.5	0.237			0.111	0.329	0.191	0.331	0.329	0.334		0.432	
S15	73	12.0% Dex-75	6.0% PEG-8000			0.01 M K/NaPB	7.4	0.5 M sucrose		0.42	1.78	0.242			0.118	0.309	0.211	0.325	0.332	0.347		1.463	
S15	74	12.0% Dex-75	6.0% PEG-8000			0.01 M K/NaPB	7.4	0.5 M trehalose		0.41	1.93	0.226			0.091	0.255	0.151	0.318	0.311	0.318		0.413	
S15	75	12.0% Dex-75	6.0% PEG-8000			0.01 M K/NaPB	7.4	0.5 M TMAO		0.42	1.37	0.233			0.208	0.505	0.2	0.255	0.304	0.768		0.431	

Ref	# ATPS	Polymer-1	Polymer-2	Salt	salt additive	Buffer	pH	1.5 M TMAO	logK HSA	logK a-Chymotrypsin	logK a-Chymotrypsinogen A	logK Con A	logK Cyt c	logK Hemoglobin bovine	logK Hemoglobin human	logK bLGA	logK bLGB	logK Lysozyme	logK Rnase A	logK Rnase B	logK Subtilisin A	logK Trypsinogen	logK Lipase
S4	1	11.10% PEG-8000		6.33% Na2SO4		0.01 M NaPB	6.8			-0.9318	-0.3675	-0.7167						-0.3915					-0.209
S4	2	11.10% PEG-10000		6.33% Na2SO4		0.01 M NaPB	6.8			-1.0343	-0.4214	-0.71						-0.3862					-0.1938
S4	3	11.10% PEG-8000		6.33% Na2SO4		0.01 M NaPB	6.8	0.5 M sorbitol		-1.2396	-0.6904	-0.7986						-0.8386					-0.2449
S4	4	11.10% PEG-8000		6.33% Na2SO4		0.01 M NaPB	6.8	0.5 M sucrose		-1.3279	-0.8041	-0.8416						-1.2596					-0.27
S4	5	11.10% PEG-8000		6.33% Na2SO4		0.01 M NaPB	6.8	0.5 M trehalose		-1.567	-0.9245	-0.9355						-1.3468					-0.2874
S4	6	11.10% PEG-8000		6.33% Na2SO4		0.01 M NaPB	6.8	0.5 M TMAO		-0.9508	-0.341	-0.7399						-0.5272					-0.2255
S2	7	6.0% PEG-8000	12.0 Dex-75		0.215M NaCl	0.01 M K/NaPB	7.4			-0.0329	0.4393	0.1706						0.3766					-0.0964

S2	8	6.0% PEG-8000	12.0 Dex-75		0.215M NaCl	0.01 M K/NaPB	7.4	0.5 M sorbitol		0.6178	0.0708	0.0682						0.5544					-0.0964
S2	9	6.0% PEG-8000	12.0 Dex-75		0.215M NaCl	0.01 M K/NaPB	7.4	0.5 M sucrose		0.7147	0.0302	0.063						0.5759					-0.0878
S2	10	6.0% PEG-8000	12.0 Dex-75		0.215M NaCl	0.01 M K/NaPB	7.4	0.5 M TMAO		-0.0146	0.4914	0.1872						0.3901					-0.0809
S2	11	6.0% PEG-8000	12.0 Dex-75		0.215M NaCl	0.01 M K/NaPB	7.4	1.5 M TMAO		0.1931	0.6484	-0.0325						0.5852					-0.1209
S2	12	6.0% PEG-8000	12.0 Dex-75		0.215M NaClO4	0.01 M K/NaPB	7.4			0.0864	0.5618	-0.6716						1.3075					-0.1385
S2	13	6.0% PEG-8000	12.0 Dex-75		0.215M NaClO4	0.01 M K/NaPB	7.4	0.5 M sorbitol		0.2133	0.8142	-0.6091						1.6665					-0.1018
S2	14	6.0% PEG-8000	12.0 Dex-75		0.215M NaClO4	0.01 M K/NaPB	7.4	0.5 M sucrose		0.266	0.7955	-0.5735						1.6911					-0.0711
S2	15	6.0% PEG-8000	12.0 Dex-75		0.215M NaClO4	0.01 M K/NaPB	7.4	0.5 M TMAO		0.1461	0.658	-0.644						1.3483					-0.1314
S2	16	6.0% PEG-8000	12.0 Dex-75		0.215M NaClO4	0.01 M K/NaPB	7.4	1.5 M TMAO		0.2049	0.8722	-0.6289						1.5899					-0.1739
S10	17	12.41 Dex-75	6.06% PEG-8000			0.01 M NaPB	7.4				0.433		-0.5376	-1.1308	-0.8827			0.3729	-0.3107	-0.342		-0.0506	-0.1451
S10	18	10.75 Dex-75	5.22% PEG-8000			0.01 M NaPB												0.2253	-0.2526				-0.1349
S10	19	8.99 Dex-75	4.34% PEG-8000			0.01 M NaPB												0.1644	-0.1637				-0.1118
S10	20	12.39 Dex-75	10.08% Ucon-4000			0.01 M NaPB	7.4				0.2504		-0.9208					0.4698	-0.6073	-0.5768		-0.1537	-0.1818
S10	21	10.42 Dex-75	8.41% Ucon-4000			0.01 M NaPB	7.4							-1.2757	-0.9318			0.4298	-0.4498				-0.1878
S10	22	8.23 Dex-75	6.82% Ucon-4000			0.01 M NaPB	7.4											0.3424	-0.27				-0.1186
S11	23	13.95% PEG-8000		8.06% Na2SO4		0.01 M UB	7.4			-1.0132	-0.6144	-1.1427			-1.0862	-2.0969	-1.2147	-0.6271	-1.5686	-1.0044	0.0508	-1.4202	
S11	24	13.95% PEG-8000		8.06% Na2SO4	0.15 M NaCl	0.01 M UB	7.4			-0.9957	-0.4895	-1.1805			-0.9469	-1.9586	-1.3665	-0.2441	-1.6383	-1.0809	0.1767	-1.4437	
S11	25	13.95% PEG-8000		8.06% Na2SO4	0.33 M NaCl	0.01 M UB	7.4			-0.9172	-0.109	-1.1612			-0.9626	-1.8239	-1.3188	0.5987	-1.5086	-1.301	0.27	-1.1487	
S11	26	13.95% PEG-8000		8.06% Na2SO4	0.15M NaSCN	0.01 M UB	7.4			-1.0315	-0.8601	-1.1487		-1.3872	-0.9393	-1.9586	-1.2924	1.1458	-1.3979	-1.0044	0.2725	-1.6198	
S11	27	13.95% PEG-8000		8.06% Na2SO4	0.40M NaSCN	0.01 M UB	7.4			-0.8041	-0.0964	-1.0555		-1.301	-0.7595	-1.8861	-1.1675	1.7435	-1.1487	-0.9747	0.3458	-1.1367	
S11	28	15.00% PEG-8000				1.4M K/NaPB	7.4		-0.4522	-0.5143		-0.8539		-0.6091	-0.3107	-0.8827	-0.8508		-0.983	-1.1612	0.3197	-0.9172	
S11	29	15.70% PEG-600		9.47% Na2SO4	0.15 M NaCl	0.058M K/NaPB	7.4		-0.4202	0.485	0.7582	-0.4724	-0.8182	0.3528	0.7027	-0.7773	-0.6055	0.8217	0.2545	-0.0721	0.2956	0.6086	
S11	30	15.70% PEG-600		9.47% Na2SO4		0.085M K/NaPB	7.4		0.0457	0.4547	0.7143	-0.4056	-1.0315	0.4014	0.8241	-0.4921	-0.399	0.7413	0.2653	-0.0209	0.2975	0.5015	
S11	31	15.70% PEG-600		9.47% Na2SO4	0.15 M NaCl	0.085M K/NaPB	7.4		-0.3726	0.6261	1.0622	-0.5607	-1.3098	0.507	0.954	-0.7932	-0.6271	1.0017	0.3727	-0.0555	0.2896	0.8333	
S11	32	15.70% PEG-600		9.47% Na2SO4	0.40M NaSCN	0.085M K/NaPB	7.4			0.9302	1.528	-0.5735	-1.699	0.7572	1.0737	-1.2147	-0.8327	1.9101	0.7005	0.0273	0.3481	1.2175	
S11	33	15.70% PEG-600		9.47% Na2SO4		0.17M K/NaPB	7.4		0.2971	0.7081	1.2458	-0.3778	-1.585	0.712	0.934	-0.5867	-0.466	1.0806	0.4317	0.0216	0.3707	0.9337	
S11	34	15.70% PEG-600		9.47% Na2SO4	0.15 M NaCl	0.17M K/NaPB	7.4		-0.1945	0.8681	1.6457	-0.3429	-2.0809	0.77		-0.8416	-0.5607	1.3659	0.5366	0.0445	0.3604	1.1801	
S11	35	15.70% PEG-600		9.47% Na2SO4	0.30 M NaCl	0.17M K/NaPB	7.4		-0.4389	1.0626	1.8844	-0.2284	-1.5376	0.7612		-0.9066	-0.7011	1.9325	0.7993	0.1052	0.3836	1.4524	
S11	36	15.70% PEG-600		9.47% Na2SO4	0.40 M NaSCN	0.17M K/NaPB	7.4			1.1587	1.4538		-1.1549	0.9567		-1.2147	-0.8268	1.9717	0.8663	0.1219	0.3849	1.8893	
S11	37	15.30% PEG-600		9.46% Na2SO4	0.15 M NaCl	0.15M K/NaPB	7.4		0.4141	0.8096	1.4994	-0.4841	-1.4318	0.6301	1.0803	-0.5952	-0.4949	0.8285	0.467	-0.0237	0.0298	1.2006	
S11	38	13.75% PEG-600				1.55M K/NaPB	7.4			1.4487			-1.4559	1.1676		1.6675	0.7093	1.8149	1.4755	0.3166	0.2033		
S12	39	9.00% PEG-8000	19.0% Ficoll-70			0.01 M K/NaPB	7.4			-0.7447	-0.2848	-0.58				-0.9031	-0.9626	-0.7305	-0.6234	-0.71			
S12	40	9.00% PEG-8000	19.0% Ficoll-70			0.01 M K/NaPB	7.4	0.5 M TMAO		-0.6968	-0.163	-0.5884				-0.7799	-0.9172	-0.767	-0.6126	-0.7212			
S12	41	9.00% PEG-8000	19.0% Ficoll-70			0.01 M K/NaPB	7.4	1.5 M TMAO		-0.58	0.1358	-0.58				-0.6696	-0.7696	-0.7471	-0.6289	-0.7986			
S12	42	9.00% PEG-8000	19.0% Ficoll-70			0.01 M K/NaPB	7.4	1.5 M TMAO		-0.4776	0.4857	-0.5735				-0.3768	-0.5058	-0.7905	-0.618	-0.8477			
S12	43	11.90% Dex-70	15.7% PEG-600		0.15 M Na2SO4	0.01 M K/NaPB	7.4		-0.2857	0.1688	0.3964	-0.6289				-0.4377	-0.4789	-0.1163	-0.0531	0.0326			
S12	44	11.90% Dex-70	15.7% PEG-600		0.15 M Na2SO4	0.01 M K/NaPB	7.4	0.5 M TMAO	-0.2013	0.2514	0.518	-0.6402				-0.4342	-0.4353	-0.1024	-0.0182	0.0745			
S12	45	11.90% Dex-70	15.7% PEG-600		0.15 M Na2SO4	0.01 M K/NaPB	7.4	1.5 M TMAO	-0.0521	0.3629	0.5776	-0.6198				-0.3363	-0.2967	-0.0458	0.0257	0.1106			
S12	46	11.90% Dex-70	15.7% PEG-600		0.15 M Na2SO4	0.01 M K/NaPB	7.4	1.5 M TMAO	0.2348	0.5031	0.7493	-0.618				-0.1018	-0.1249	0.0128	0.1082	0.185			
S12	47	11.90% Dex-70	15.7% PEG-600		0.15 M Na2SO4	0.01 M K/NaPB	7.4	1.95 M TMAO	0.5921	0.5997	0.8794	-0.5986				0.1079	0.1133	0.0645	0.1778	0.2395			
S13	48	12.4% Dex-70	6.1% PEG-8000		0.15 M NaCl	0.01 M NaPB	7.4				0.433		-0.5376	-1.1308	-0.8827			0.3729	-0.3107	-0.342		-0.0506	-0.1451
S13	49	12.4% Dex-70	10.1% Ucon-4000		0.15 M NaCl	0.01 M NaPB	7.4				0.2504		-0.9208	-1.2757	-0.9318			0.4698	-0.6073	-0.5768		-0.1537	-0.1818
S13	50	15.0% PEG-8000	30.0% Ucon-4000		0.15 M NaCl	0.01 M NaPB	7.4				-2.0088							-1.4437	-1.8539			-1.8239	-0.4461
S13	51	15.10% Ficoll-70	7.90% PEG-8000		0.15 M NaCl	0.01 M NaPB	7.4				0.017			-1.0269	-0.8153			-0.041	-0.3316	-0.3565		-0.2366	-0.1349
S13	52	13.00% Ficoll-70	9.93% Ucon-4000		0.15 M NaCl	0.01 M NaPB	7.4				-0.1952			-1.284	-1.1308			0	-0.6021	-0.6253		-0.4622	-0.1979
S13	53	15.2% PES-100	6.96% PEG-8000		0.15 M NaCl	0.01 M NaPB	7.4				0.4742			-0.8297	-0.6576			0.1106	-0.219	-0.153		-0.0146	-0.1249
S13	54	12.9% PES-100	7.68% Ucon-4000		0.15 M NaCl	0.01 M NaPB	7.4				0.2553			-0.6819	-0.5498			0.1072	-0.2958	-0.2007		-0.1085	-0.1079
S1	55	11.10% PEG-8000		6.33% Na2SO4	0.215M NaCl	0.01 M NaPB	6.8			-0.9586	-0.2652	-0.7375						-7.45E-03					-0.2336
S1	56	11.10% PEG-10000		6.33% Na2SO4	0.215M NaCl	0.01 M NaPB	6.8			-1.0894	-0.3261	-0.762						-0.032					-0.2328
S1	57	11.10% PEG-8000		6.33% Na2SO4	0.215M NaCl	0.01 M NaPB	6.8	0.5 M sorbitol		-1.3851	-0.475	-0.8356						-0.2749					-0.2725
S1	58	11.10% PEG-8000		6.33% Na2SO4	0.215M NaCl	0.01 M NaPB	6.8	0.5 M sucrose		-1.3665	-0.4377	-0.8861						-0.475					-0.2823
S1	59	11.10% PEG-8000		6.33% Na2SO4	0.215M NaCl	0.01 M NaPB	6.8	0.5 M TMAO		-1.0883	-0.2211	-0.7773						-0.1046					-0.2692
S14	60	12.33% Dex-69	6.05% PEG-8000			0.01 M NaPB	7.4		-1.1192	-0.2366	0.066	-0.9031	-1.1739	-0.8013	-0.6108	-0.426	-0.4134	-0.6478	-0.6478	-0.2557	0.0558	-0.3747	
S14	61	12.33% Dex-69	6.05% PEG-8000		0.15 M NaCl	0.01 M NaPB	7.4			-0.066	0.3838	-0.8761	-0.6737	-0.8665	-0.684	-1.1427	-0.8601	0.2876	-0.2343	-0.2299	0.167	-0.0904	
S14	62	12.33% Dex-69	6.05% PEG-8000		1.05 M NaCl	0.01 M NaPB	7.4			0.129	0.8267	-0.821	-0.6383	-0.5735	-0.4498	-1.2676	-0.5918	1.017	-0.1209	-0.1911	0.306	0.1912	
S14	63	12.33% Dex-69	6.05% PEG-8000		0.083M Na2SO4	0.01 M NaPB	7.4		-1.6021	1.4487	0.2156	-0.8297	-1.2676	-0.8356	-0.7399	-1.0269	-0.7905	-0.3458	-0.4908	-0.4134	0.1199	-0.3089	
S14	64	12.33% Dex-69	6.05% PEG-8000		0.30 M Na2SO4	0.01 M NaPB	7.4			-0.2262	0.3574	-0.8508	-1.3979	-0.9101	-0.7799	-1.301	-0.9101	-0.1624	-0.3969	-0.4989	0.1973	-0.2373	
S14	65	12.33% Dex-69	6.05% PEG-8000		0.17 M CsCl	0.01 M NaPB	7.4			-0.1051	0.386	-0.8761	-0.7011	-0.9318	-0.7471	-1.1938	-0.8665	0.3788	-0.2573	-0.2034	0.0881	-0.1537	
S14	66	12.33% Dex-69	6.05% PEG-8000		0.80 M CsCl	0.01 M NaPB	7.4			0.0484	0.7324	-0.8508	-0.7055	-0.7545	-0.5817	-1.2757	-0.6421	0.8745	-0.1838	-0.2708	0.2198	-0.0535	
S14	67	12.33% Dex-69	6.05% PEG-8000		0.094 M NaClO4	0.01 M NaPB	7.4			-0.0315	0.5626	-0.8447	-0.4597	-0.8416	-0.7471	-1.2518	-0.7399	1.1644	-0.1261	-0.2644	0.1313	0.012	
S14	68	12.33% Dex-69	6.05% PEG-8000		0.43 M NaClO4	0.01 M NaPB	7.4			0.029	0.7193	-0.821	-0.4318	-0.7167	-0.5784	-1.284	-0.9172	1.7267	-0.1129	-0.2373	0.2114	1.30E-03	
S14	69	12.33% Dex-69	6.05% PEG-8000		0.17 M NaSCN	0.01 M NaPB	7.4			-2.18E-03	0.5877	-0.8386	-0.4437	-0.8729	-0.6162	-1.2676	-0.9281	1	-0.0975	-0.2373	0.1711	-5.68E-03	
S14	70	12.33% Dex-69	6.05% PEG-8000		1.26 M NaSCN	0.01 M NaPB	7.4			0.0141	0.6201	-0.767	-0.6038	-0.5072	-0.2857	-1.1938	-0.6819	1.3617	-0.1391	-0.1818	0.1928	-0.1107	
S15	71	12.0% Dex-75	6.0% PEG-8000			0.01 M K/NaPB	7.4			-0.3768	0	-0.6271			-0.8996	-0.3372	-0.4815	-0.6383	-0.5045	-0.1073		-0.4473	
S15	72	12.0% Dex-75	6.0% PEG-8000			0.01 M K/NaPB	7.4	0.5 M sorbitol		-0.3696	0.1761	-0.6253			-0.9547	-0.4828	-0.719	-0.4802	-0.4828	-0.4763		-0.3645	
S15	73	12.0% Dex-75	6.0% PEG-8000			0.01 M K/NaPB	7.4	0.5 M sucrose		-0.3768	0.2504	-0.6162			-0.9281	-0.51	-0.6757	-0.4881	-0.4789	-0.4597		0.1652	
S15	74	12.0% Dex-75	6.0% PEG-8000			0.01 M K/NaPB	7.4	0.5 M trehalose		-0.3872	0.2856	-0.6459			-1.041	-0.5935	-0.821	-0.4976	-0.5072	-0.4976		-0.384	
S15	75	12.0% Dex-75	6.0% PEG-8000			0.01 M K/NaPB	7.4	0.5 M TMAO		-0.3768	0.1367	-0.6326			-0.6819	-0.2967	-0.699	-0.5935	-0.5171	-0.1146		-0.3655	

Table S3. Logarithms of distribution coefficients for various drugs in octanol-buffer systems of various ionic compositions (NaPB – sodium phosphate buffer, UB - Universal buffer composed of 0.01M or 0.10M each of phosphoric, boric, and acetic acids adjusted to pH 7.4 with NaOH)

Component-1	Component-2	logD 5-Hydroxytryptophan	logD Acebutolol HCl	logD a-Methyldopa	logD Amoxicillin	logD Atenolol	logD Carbamazepine	logD Chlorpromazine HCl
	0.01M NaPB	-1.395	-0.09	-2.14	-3.18	-0.21	1.48	2.03
	0.01M UB	-2	0	-2.35	-3.4	-0.18	1.34	2.2
	0.10M NaPB	-1.95	0.04	-2.06	-3.05	-1.43	1.47	3.25
	0.10M UB	-2.09	0.46	-3.19	3.18	-0.81	1.39	3.38
0.15M NaCl	0.01M NaPB	-2.03	-0.19	-2.24	-3.37	-0.08	1.44	2.35
0.15M NaCl	0.01M UB	-2.05	-0.1	-2.28	-3.38	-0.25	1.37	2.17
0.15M NaCl	0.10M NaPB	-1.98	0.08	-2.13	-3.11	-1.38	1.49	3.18
0.15M NaCl	0.10M UB	-2.07	0.4	-3.09	-3.21	-0.85	1.41	3.49

Component-1	Component-2	logD Clonidine HCl	logD Desipramine HCl	logD Diclofenac Na	logD Doxepin HCl	logD Furosemide	logD Harmine	logD Hydrochlorocyclizine 2HCl
	0.01M NaPB	0.84	0.81	0.05	1.41	-1.77	2.51	0.43
	0.01M UB	0.91	0.85	-0.04	1.48	-1.86	2.47	0.92
	0.10M NaPB	1.2	1.32	0.69	2.46	-1.58	2.51	2.7
	0.10M UB	1.34	1.7	0.6	2.62	-1.59	2.48	2.88
0.15M NaCl	0.01M NaPB	0.64	1.03	0.62	1.47	-1.46	2.51	1.43
0.15M NaCl	0.01M UB	0.66	1.01	0.55	1.35	-1.51	2.45	1.36
0.15M NaCl	0.10M NaPB	1.13	1.4	0.85	2.4	-1.47	2.53	2.67
0.15M NaCl	0.10M UB	1.29	1.6	0.78	2.62	-1.49	2.49	2.8

Component-1	Component-2	logD Hydrochlorothiazide	logD Imipramine	logD Indomethacin	logD Maprotiline HCl	logD Mefexamide HCl	logD Metoprolol (1/2 tartrate)	logD Minaprine 2HCl
	0.01M NaPB	-0.37	1.5	0.71	0.83	0.37	-0.33	-0.46
	0.01M UB	-0.48	1.6	0.63	0.88	0.43	-0.19	0.37
	0.10M NaPB	-0.35	2.62	0.63	1.28	0.85	0.03	2.04
	0.10M UB	-0.5	2.84	0.55	1.63	1.21	0.47	2.03
0.15M NaCl	0.01M NaPB	-0.42	1.69	1.02	1.22	0.25	-0.3	0.05
0.15M NaCl	0.01M UB	-0.5	1.58	0.94	1.13	0.29	-0.2	0.38
0.15M NaCl	0.10M NaPB	-0.37	2.58	0.81	1.42	0.78	0.11	1.99
0.15M NaCl	0.10M UB	-0.53	2.85	0.74	1.65	1.06	0.39	2.01

Component-1	Component-2	logD Piroxicam	logD Propranolol	logD Sulfamethizole	logD Terbutaline	logD Theophylline	logD Thioridazine	logD Verapamil
	0.01M NaPB	-0.31	0.71	-1.07	-1.23	0.08	2.07	1.65
	0.01M UB	-0.39	0.77	-1.18	-1.09	0.11	2.4	1.79
	0.10M NaPB	-0.83	1.39	-2.18	-1.33	0.13	3.45	2.79
	0.10M UB	-0.9	1.67	-2.48	-0.87	0	3.6	3.03
0.15M NaCl	0.01M NaPB	-0.24	0.84	-1.15	-1.29	0.13	2.56	1.79
0.15M NaCl	0.01M UB	-0.21	0.83	-1.16	-1.2	0.11	2.49	1.67
0.15M NaCl	0.10M NaPB	-0.73	1.35	-2.17	-1.24	0.15	3.4	2.76
0.15M NaCl	0.10M UB	-0.79	1.56	-2.33	-0.92	0.02	4.33	3.04

Table S4. Logarithms of partition coefficients of drugs between blood and various tissues in rats in vivo (data from [S16])

Tissues/Drug	5-butyl-5-ethyl barbituric acid	5-heptyl-5-ethyl barbituric acid	5-hexyl-5-ethyl barbituric acid	5-methyl-5-ethyl barbituric acid	5-nonyl-5-ethyl barbituric acid	5-octyl-5-ethyl barbituric acid	5-pentyl-5-ethyl barbituric acid	5-propyl-5-ethyl barbituric acid
Muscle	-0.0706	0.1818	0.2553	-0.1427	0.0899	0.0453	0.0414	0.0969
Bone								
Brain	-0.0655	0.0864	0.2455	-0.2007	0.42	0.3444	-0.1612	-0.0223
Heart	0.0128	0.1492	0.2279	-0.1612	0.0828	0.1761	0.0294	4.32E-03
Intestine	-8.77E-03	0.1038	0.2068	-0.2366	0.3222	0.1673	-0.0506	-0.0757
Skin	-0.1079	0.2068	0.3444	-0.0862	0.5775	0.5145	-0.1427	0.0334
Lungs	-0.1024	0.0453	0.0334	-0.1308	0.5539	0.5276	-0.1192	0.0531
Spleen	-0.2366	0.0453	0	-0.1938	0.4983	0.3032	-0.2441	-0.0809
Adipose	-0.0132	0.7959	0.8859	-0.6021	0.8627	0.7882	0.2304	-0.0605
Liver	0.1931	0.4249	0.525	0.1959	0.6758	0.7284	0.2095	0.29
Kidney	0.3784	0.3263	0.356	0.1038	0.3139	0.444	0.2279	0.4713
Stomach	-0.0362	0.4857	0.4786	-0.585	0.5478	0.2788	0.017	-0.2076
Pancreas	-0.3188	0.2068	0.2068	-0.3098	0.4609	0.4116	-0.1192	-0.1612

Tissues/Drug	Acebutolol	Alfentanyl	Alprazolam	Barbital	Betaxolol	Biperiden	Bisoprolol	Carvedilol
Muscle	0.6513	-0.2924	0.3385	-0.0862	0.8376	0.5224	0.5933	0.1206
Bone		-1.3979				0.8169	0.2923	0.5328
Brain	-0.4089	-0.6383	0.1987	-0.1739	0.8156	0.8382	0.0969	
Heart	0.6758	-0.041		-0.1367	1.0531	0.8463	0.683	0.7853
Intestine	1.933	0.601	0.1399	-0.2291	1.2923	1.017	1.281	
Skin	0.415	-0.4685	0.281	-0.0809	0.4997	0.5809	0.2068	
Lungs	0.8899	0.1106		-0.0706	1.9877	1.7528	1.4886	
Spleen		0.1335		-0.1938				0.8222
Adipose	-0.0757	0.5988	4.32E-03	-0.3468	0.1644	1.7796	-0.1249	
Liver	1.4265	0.2742	0.8463	0.3385	1.7796		1.2253	0.932
Kidney	1.4314	0.1903	0.4249	0.2279	1.4533	1.0086	1.2601	0.716
Stomach				-0.5528				
Pancreas		0.2553		-0.3279				

Tissues/Drug	Cefazolin	Ceftazidime	Chlordiazepoxide	Cisapride	Cotinine	Decane	Deltamethrin	Diazepam
Muscle	-0.6576	-0.7212	-0.2596		-0.1612	0.7202	1.5159	0.3541
Bone		-0.6383						
Brain			-0.2757	0.1461	-0.3468	1.0334	0.1106	0.3096
Heart	-0.7212	-0.6576	0.2718	0.238	-0.2924			0.6937
Intestine	-0.4089	-0.3872	0.1492		-0.2218		0.4116	0.5911
Skin	-0.2007	-0.4089	-0.4685					0.7332
Lungs	-0.3768	-0.3565		0.9877	-0.2366			0.6599
Spleen								
Adipose		-0.7959	0.49		-1.0969	1.4216	2.4579	1.415
Liver	0.2788	-0.6021	0.5416	1.1875	-0.1938	1.0334	0.4116	0.7966
Kidney	0.8267	0.6884	0.2878	0.8189	-4.36E-03			0.6618
Stomach								
Pancreas								

Tissues/Drug	Dicloxacillin	Dideoxyinosine	Digoxin	Domperidone	Enoxacin	Etodolac	Fentanyl	Fingolimod
Muscle	-1.301	-0.1612	0.0828	0.4298	0.1875		0.5635	1.0212
Bone						0.1847		
Brain		-0.7696		-1.0458		-1.0458	0.6212	1.692
Heart	-1.1549		0.2068	0.48	0.0531	-0.1938	0.7275	1.2405
Intestine		-0.2924	0.7543				1.0128	
Skin					0.1584		0.4116	
Lungs	-0.9208		0.3032	0.9009	0.0828		1.2041	1.8338
Spleen	-1.0458	-0.1739			0.238		1.534	1.7931
Adipose						-0.6198	1.5198	
Liver		-0.1192	1.1644	1.0294	0.5328	-0.301	0.6532	1.6721
Kidney		0.8357	0.2967	1.243	0.6902	-0.3188	1.1553	1.5539
Stomach								
Pancreas		-0.1487					1.4216	

Tissues/Drug	Fleroxacin	Flunitrazepam	Ftorafur	Galanthamine	Glycyrrhetinic acid	Glycyrrhizin	Hexobarbital	Imipramine
Muscle	0.1903	-0.1192	-0.0458	0.3118	-0.7447	-1	-0.1024	0.7218
Bone		-0.0315						
Brain		0.1367	-0.1367	0.1614	-1.1549			1.1038
Heart	0.2945	-0.0223	-0.1612		-0.6778	-1.3979	0.1139	1.1173
Intestine		0.601	-0.1871				0.1523	1.1461
Skin			-0.1427		-0.5376	-0.5528	-0.0362	
Lungs	0.2201		-0.3372		-0.3979	-1	0.4487	1.8825
Spleen			-0.1192		-0.8861			1.5353
Adipose		1.7723	-0.5086				0.2148	0.7987
Liver		0.48	-0.1549	0.3856			0.7752	1.4914
Kidney		-0.4815	0.0899	1.1239			0.179	1.4362
Stomach								
Pancreas			-0.4437					1.4166

Tissues/Drug	Inaperisone	Laniquidar	Lidocaine	Lomefloxacin	Lorcainide	Lubeluzole	Matrine	Metoprolol
Muscle	0.3385	0.9513	0.1206	0.2253	0.3711	0.4281	0.1271	0.5682
Bone				0.2148				0.5391
Brain	0.8156	0.5587	0.4065	-0.6576	0.1004	0.7348	0.3139	0.6464
Heart	0.5944	0.8669	0.3304	0.1523	0.382		0.1875	0.6365
Intestine			0.3892	0.2279			0.49	0.902
Skin	0.525		0.3075	-0.0132				0.3032
Lungs	1.248	1.6893	0.4757	0.1106	1.2068	1.3766	0.1875	1.2355

Spleen			0.5763	0.2553			0.8122	
Adipose	0.9159			-0.5686			-0.3372	-0.1805
Liver	1.2553	1.3263	0.9571	0.3784	-0.3279	1.5611	0.7536	1.4456
Kidney	1.4886	1.179	2.1303	0.7024	0.6739	1.1139	1.0128	1.2455
Stomach								
Pancreas								

Tissues/Drug	Midazolam	Miloxacin	Molinate	Nalidixic acid	Nebivolol	Nicotine	o-Ethoxybenzamide	Ofloxacin
Muscle	0.1139		0.6201	-0.4559	0.3617	0.2175	-0.1024	0.2695
Bone				-0.5528				0.1875
Brain	0.4757	-0.9208		-0.6778	0.4639	0.4378	-0.0269	-0.6021
Heart	0.4814			-0.3188	0.5647	0.2355	0	0.2856
Intestine	0.4829			-0.3188		0.301	-0.2441	
Skin	0.4249			-0.4685		0.1072	4.32E-03	0.1106
Lungs	0.5132	-0.2924		-0.4949	1.891	0.243	-0.0362	0.1673
Spleen	0.3692	-0.4815		-0.4815			-0.0655	0.3201
Adipose	0.776		2.2504	-1.0458		-0.3279	-0.1549	-0.699
Liver	1.0531		1.1492	-0.2441	1.0414	0.6821		0.3444
Kidney	0.5079			-0.2757	0.918	1.2504	0.1106	0.8414
Stomach	0.4871							
Pancreas								

Tissues/Drug	Oxprenolol	Pefloxacin	Penicillin	Pentazocine	Perchlorethylene	Phencyclidine	Phenobarbital	Phenytoin
Muscle	0.6551	0.4099	-0.9586	0.6149	0.2648	0.1303	-0.0862	-0.0969
Bone	0.4409			0.5798				
Brain	0.3747	-0.7696		0.4829	0.4728	0.3598		-0.1427
Heart	0.7218	0.3979	-0.7696	0.5798		0.29	-0.0915	-0.0969
Intestine	1.2095		0.243	0.5119			0.1072	0.1903
Skin	0.3032			0.5119			4.32E-03	0.0414
Lungs	1.3838	0.3139	-0.5528	1.2788		1.5623	-0.2007	-0.1249
Spleen		0.5599	-0.7696					
Adipose	-0.0809			0.2405	2.0719	1.7396	-0.6383	0.2175
Liver	1.1303	0.7543	-0.3468	0.243	0.4099	0.8555	0.1818	0.3579
Kidney	1.3139	0.6425	0.828	1.1818	0.3802	1.0212	-0.2147	0.1987
Stomach								
Pancreas								

Tissues/Drug	Pindolol	Pipemidic acid	p-Phenylbenzoic acid	Procainamide	Propanolol	Prucalopride	Pseudocumene	Quinidine
Muscle	0.7745	0.1461	-0.8539	0.49	0.6628	0.4771	0.0828	0.4857
Bone	0.29	0.3784			0.6739			
Brain	0.5944	-0.8861	-1		1.238	-0.5528	0.4031	-0.1938
Heart	0.9652	0	-0.3872	0.3909	0.9657	0.4502		0.617
Intestine	1.243		-0.5686		1.2833			0.8579
Skin	0.3324		-0.5686		0.5623			
Lungs	1.3927	0.0682	-0.301		1.7619	0.8432		1.4857
Spleen		0.1875	-0.7447					1.2304
Adipose	-0.2218	-0.4437		-0.9208	0.2989		1.7973	
Liver	0.9455	0.6893		0.5011	1.29	0.7604	0.4031	1.0682
Kidney	1.49	0.8965		0.8028	1.1303	1.0607		1.1673
Stomach								
Pancreas								

Tissues/Drug	Ritanserin	Sabeluzole	Salicylic acid	Styrene	Sufentanyl	Tenoxicam	Tetracycline	Theophylline
Muscle	0.6075		-0.6021	-0.0655	0.3927	-1.0969	0.301	-0.2147
Bone			-0.585			-1	0.9504	
Brain	0.4698	0.8055	-1.0458		0.4771	-2		-0.4437
Heart			-0.4685		0.415	-0.7447		
Intestine			-0.4202			-0.699	0.574	
Skin			-0.3098			-0.8239		
Lungs	1.5079	1.5403	-0.4559	-0.1938	0.9509	-0.5086		-0.1427
Spleen						-1.1549		
Adipose				1.6117		-1.5229	0.0414	
Liver	1.3979	1.6513	-0.3768	0.0719	-0.1739	0.0607	0.6721	
Kidney	1.1492	1.0899	-0.0915		0.2279	0.017	0.6075	
Stomach								
Pancreas						-0.9208		

Tissues/Drug	Thiopental	Timolol	Tolbutamide	Triazolam	Valproic acid	Verapamil	Zearalenone
Muscle	-0.1427	0.5763	-0.8239	0.6064	-0.6778	0.6138	-0.3665
Bone		-0.0458					
Brain	-0.1308	-0.0177	-0.9586		-0.4685		0.0253
Heart	0.0792	0.6866	-0.4815		-0.2366	0.8482	0.0645
Intestine	0.1335	1.2625	-0.8539	0.902	-0.2147		1.5775
Skin	0.1492	0.1553	-0.5528	0.5635	-0.1938		
Lungs	0.1239	1.3892	-0.4815		-0.2441	1.7694	0.3636
Spleen	-0.2147		-0.6576				-0.0915
Adipose	0.8169	-0.2366	-0.8539	0.6064	-0.699		0.5198
Liver	0.5302	0.8579	-0.7696	0.3997	0.3909		0.659
Kidney	0.4133	1.0828	-0.7959	0.752	0.3118	1.1673	-0.2596
Stomach							0.1139
Pancreas	0.0253		-0.7447				
